# A stable 15-member bacterial SynCom promotes *Brachypodium* growth under drought stress

**DOI:** 10.3389/fmicb.2025.1649750

**Published:** 2025-08-11

**Authors:** Archana Yadav, Mingfei Chen, Shwetha M. Acharya, Grace Kim, Yuguo Yang, Tiffany Z. Zhao, Eunice Tsang, Romy Chakraborty

**Affiliations:** ^1^Climate and Ecosystem Sciences Division, Earth & Environmental Sciences Area, Lawrence Berkeley National Laboratory, Berkeley, CA, United States; ^2^Department of Molecular and Cell Biology, University of California Berkeley, Berkeley, CA, United States

**Keywords:** microbiome, rhizosphere, *Brachypodium*, drought, SynCom, plant growth promotion (PGP)

## Abstract

**Introduction:**

Rhizosphere microbiomes are known to drive soil nutrient cycling and influence plant fitness during adverse environmental conditions. Field-derived robust Synthetic Communities (SynComs) of microbes mimicking the diversity of rhizosphere microbiomes can greatly advance a deeper understanding of such processes. However, assembling stable, genetically tractable, reproducible, and scalable SynComs remains challenging.

**Methods:**

Here, we present a systematic approach using a combination of network analysis and cultivation-guided methods to construct a 15-member SynCom from the rhizobiome of *Brachypodium distachyon*. This SynCom incorporates diverse strains from five bacterial phyla. Genomic analysis of the individual strains was performed to reveal encoded plant growth-promoting traits, including genes for the synthesis of osmoprotectants (trehalose and betaine) and Na^+^/K^+^ transporters, and some predicted traits were validated by laboratory phenotypic assays.

**Results:**

The SynCom demonstrates strong stability both *in vitro* and *in planta*. Most strains encoded multiple plant growth-promoting functions, and several of these were confirmed experimentally. The presence of osmoprotectant and ion transporter genes likely contributed to the observed resilience of *Brachypodium* to drought stress, where plants amended with the SynCom recovered better than those without. We further observed preferential colonization of SynCom strains around root tips under stress, likely due to active interactions between plant root metabolites and bacteria.

**Discussion:**

Our results demonstrate that trait-informed construction of synthetic communities can yield stable, functionally diverse consortia that enhance plant resilience under drought. Preferential colonization near root tips points to active, localized plant–microbe signaling as a component of stress-responsive recruitment. This stable SynCom provides a scalable platform for probing mechanisms of plant-microbe interaction and for developing microbiome-based strategies to improve soil and crop performance in variable environments.

## Introduction

It is widely acknowledged that root-associated microorganisms play a crucial role in plant health and productivity. Root microbiomes aid plants by promoting nutrient acquisition (Mendes et al., 2013), producing plant growth hormones (Dodd et al., 2010; Eichmann et al., 2021), and conferring resilience under abiotic stresses such as drought and increase in salinity (Layeghifard et al., 2017; Schmitz et al., 2022) among other beneficial functions. Plants recruit these root microbiomes primarily through the secretion of root exudates, that include organic acids, amino acids, nucleotides, vitamins, and fatty acids (Kawasaki et al., 2016; Zhalnina et al., 2018; Berendsen et al., 2018), orchestrating over time the structure of their root-associated microbiome (Voges et al., 2019; Pascale et al., 2019).

Numerous studies have emphasized the importance of root microbiomes (Bulgarelli et al., 2015; Liu et al., 2021), and have shown that specific microbial groups play a more crucial role than others (Gkarmiri et al., 2017; Bergelson et al., 2019). However, despite recent advancements in the field, identifying and selecting beneficial root microbes for applications in crop improvement continues to remain challenging, since a holistic understanding of the complex interactions between microbes and their host plants is still lacking (Finkel et al., 2017; Diwan et al., 2022; Kimotho and Maina, 2024). One promising approach involves constructing simplified synthetic communities (SynComs) derived from the root microbiome, where individual microbial strains co-exist, interacting with one another (Gupta et al., 2021; Shayanthan et al., 2022). Defined SynComs enable deeper understanding of how microbes cooperate, compete, and influence each other and the plant host in the rhizosphere ecosystem (de Souza et al., 2020), and allow designing targeted experiments to generate defensible hypotheses about role of microbial communities during plant growth and health under myriad environmental conditions. SynComs are typically constructed using either a reductionist approach or holistic approach. The reductionist approach relies on cultivation-based techniques, where microbes are isolated and cultured in the laboratory for study (Vorholt et al., 2017; Rodríguez Amor and Dal Bello, 2019; Liu et al., 2019; Xia et al., 2020). In contrast, the holistic approach assembles SynComs based on key microbial interactions observed in natural environment using culture-independent techniques like high-throughput sequencing (Zegeye et al., 2019; Gonçalves et al., 2023) However, both approaches have limitations. The reductionist approach often excludes key uncultivable microbes or assembles strains that do not originate from the rhizosphere microbiome, and therefore have genotypic and functional differences compared to microbes naturally present in rhizosphere soil (Liu et al., 2019). In contrast, the holistic approach relies heavily on genome sequencing, fails to differentiate between active or inactive bacteria—potentially leading to inaccurate diversity estimates—and assumes that the presence of genomic signatures directly correlates with functional activity (Gupta et al., 2021; Gutleben et al., 2018). These limitations highlight the need for an integrative strategy to construct SynComs that better mimic natural microbe-microbe and microbe-plant interactions (Chodkowski and Shade, 2017; McCarty and Ledesma-Amaro, 2019).

For broader applicability, it is desirable that a SynCom is stable (with robust colonization and prevalence throughout plant development), effective (able to confer beneficial traits to plants), and reproducible (yield consistent results across laboratory experiments). However, the successful assembly of such SynComs has been limited to a handful of prior reports (Shayanthan et al., 2022; McCarty and Ledesma-Amaro, 2019). In addition, only a few studies (Sanchez-Gorostiaga et al., 2018; Toju et al., 2020; Kabir et al., 2024) have demonstrated the persistence of introduced SynCom members in the rhizosphere, highlighting a critical gap in understanding their long-term functionality. Also, there is a growing interest in exploring the ability of rhizosphere microbiomes to alleviate drought stress in host plants observeable through declining crop production (Orimoloye, 2022; Tp, 2023; Seleiman et al., 2021). Similarly, poor agricultural practices like high-salinity irrigation is leading to salt stress, further intensifying crop yield losses (Atta et al., 2023). As a result, developing effective and stable SynComs—particularly those that enhance plant resilience under these stress conditions has emerged as a key focus in recent research. Syncoms offer a natural and sustainable alternative to over-reliance on chemical fertilizers for improving crop productivity (Shayanthan et al., 2022; Hao et al., 2021), though they have been addressed in only a limited number of studies (Schmitz et al., 2022; Flores-Duarte et al., 2023).

In this study, we hypothesize that a carefully designed and assembled SynCom, derived from the naturally grown *Brachypodium* rhizobiome, can enhance and support plant growth during environmental stress of drought and increased salinity. Using a combinatorial approach, we integrated culture-independent rhizobiome community analysis with network analysis to uncover critical microbe-microbe interactions. We then used culture-dependent methods to isolate key highlighted microbial strains, ultimately constructing a 15-member SynCom. We tested the stability and effectiveness of this SynCom. Genomic and phenotypic characterization of individual strains confirmed presence of plant-growth-promoting traits, and the SynCom’s persistence was demonstrated when grown in planta. To further evaluate its effectiveness, we amended *Brachypodium* seedlings with the SynCom and subjected them to salinity and drought stresses. Using 16S amplicon sequencing, whole genome sequencing, assays for plant-growth-promoting traits, and plant phenotyping, we investigated correlations between plant phenotypes, SynCom abundance, and the SynCom’s beneficial effects under these stress conditions. Finally, we examined the spatial distribution of SynCom strains on roots to determine whether they preferentially colonize specific root niches, providing insights into plant-microbe interactions under stress.

## Materials and methods

### Microbial isolation and identification

The 15-member SynCom was created using results from two interconnected experiments. In the first study (Acharya et al., 2023b), the microbe recruitment along the root surface was studied in young *Brachypodium* plants grown in natural soil. In the subsequent study (Chen et al., 2024), over 750 stable reduced community consortia (RCC) were enriched by growing root-attached microbes from the previous study over either 3 or 7 days in media with carbon substrates generally present in *Brachypodium* root exudates. In this study, based on high species diversity as denoted by the richness and Shannon diversity index, enrichments grown on two media, i.e., 0.1X R2A (BD Diagnostics), and RCH2 minimal media (Chakraborty et al., 2017) supplemented with carbon sources (either Glutamine or mixed carbon), were used to isolate colonies on the corresponding agar media plates by incubating the plates at 30°C in the dark for 7 days. Morphologically distinct colonies were picked and streaked for further purification. For species identification of the isolates, genomic DNA extracted using a PureLink Genomic DNA Mini Kit (Invitrogen, United States), was amplified using the universal 16S rRNA eubacterial primer pairs, i.e., 8F/27F and 1492R. DNA sequencing was carried out at the UC Berkeley DNA Sequencing Facility. Geneious Prime v2020.2.5 was used to process the 16S reads and the resulting consensus sequences were taxonomically classified using the SILVA database (Gurevich et al., 2013).

### Genomic analysis of individual isolates

The whole genome sequencing was performed at Novogene (Illumina Novaseq 6,000 platform). Genome assembly and annotation were performed using the KBase platform (Arkin et al., 2018). Raw reads quality was assessed with FastQC v0.11.9, followed by trimming using Trimmomatic v0.36 (Bolger et al., 2014). The reads were then assembled using Spades v3.15.3 (Prjibelski et al., 2020) and the genome quality was evaluated using CheckM v1.0.18 (Parks et al., 2015). DRAM v0.1.2 (Shaffer et al., 2020) and Blastkoala (Kanehisa et al., 2016) were used to assign KEGG orthology (KO) numbers and functional annotation to the protein-coding genes and KEGG mapper (Kanehisa and Sato, 2020) was used to visualize metabolic pathways. Annotations from the metabolic assembly output files obtained from DRAM and KEGG were used to search for specific properties or genes such as PGP traits, transporters, and osmoprotectant-related genes. For the taxonomic classification, a concatenated alignment of 120 single-copy marker proteins was created using the GTDB-tk workflow (Parks et al., 2022; Chaumeil et al., 2022). Subsequently, 4–5 representative sequences closest to the SynCom strains were selected and aligned using clustalo (Sievers et al., 2011). This was followed by the tree construction with FastTree (Price et al., 2009) and visualization using iTOL (Letunic and Bork, 2007).

### Testing persistence of SynCom members under *in vitro* conditions

We evaluated *in vitro* stability of the 15-member SynCom by tracking changes in the relative abundances of the strains over a three-week period. For this, we cultured 5 mL of the inoculum, containing equal cell numbers (4×10^7^ cells) of each SynCom member, in 45 mL of 0.2X MS (Murashige and Skoog) media (M0404, Sigma Aldrich, United States). The mixture was incubated in a plant growth chamber with conditions set at 16-h light, 24°C temperature, and 50% humidity. At the end of each week, 5 mL of the culture was transferred to 45 mL of fresh 0.2X MS media, and 5 mL of the culture was sampled for 16S rRNA community analysis.

### Seed germination and preparation of inoculum for in planta experiments

Approximately 150 *Brachypodium distachyon* (Bd 21–3) seeds were dehusked and sterilized by following the procedures as described in the protocol (Novak et al., 2024). Seeds were immersed in 70% ethanol for 30 s, 50% bleach for 5 min, and then washed five times with autoclaved MQ water. The sterilized seeds were stored in sterile water at 4°C in the dark for 1 week. Subsequently, the seeds were germinated on semisolid plates containing 0.5X MS media with 0.4% w/v phytagel and placed in a plant growth chamber (Percival Scientific AR-41 L3, United States) set to 16 h of light, 24°C temperature, and 50% humidity for 3–5 days (Ingram et al., 2012; Li et al., 2019). For potting, sterilized plastic pots (3.9”D x 3.94” W x 3.15” H, Manufacturer: MiMiLai) lined with coffee filters and filled with autoclaved calcined clay (PROFILE Products LLC, United States) were used. The clay was saturated with 0.2X MS media before transplanting one seedling per pot. The weight of each pot, once filled with clay and plants and watered to clay saturation, was recorded as the saturation weight.

Individual SynCom isolates were cultivated in R2A media for 24–72 h until they reached log phase (Supplementary Figure S8). After cultivation, the isolates were checked for purity, washed three times, and resuspended in a 30 mM phosphate buffer. For each isolate suspension, cells were stained with the nucleic acid stain SYBR green and counted using a flow cytometer (AttuneNxT®, ThermoFisher Scientific) following the manufacturer’s instructions. Suspensions for individual isolates were adjusted to equal cell numbers, specifically 4.1 × 10^7^ cells, for the final inoculum used in subsequent *in planta* experiments.

### Assessing the performance of SynCom under drought and salinity stress

This experiment comprised three conditions: Drought, Rewatered drought, and Salinity, to reflect natural environmental stresses. Each condition included two experimental sets; (1) SynCom-amended plants where 1 mL of 30 mM phosphate buffer containing equal cell numbers of each SynCom strain were inoculated at the base of plant shoot 3 days before inducing stress conditions, and (2) unamended plants where 1 mL of 30 mM phosphate buffer was inoculated at the base of plant shoot 3 days before inducing stress conditions. Each experimental set contained 7 plant replicates. After transplanting seedlings into pots, they were watered with 0.2X MS media to maintain 80% saturation weight for 4 days, after which they were inoculated with either SynCom or buffer, as explained earlier.

In addition to the three stress conditions, there was a control set with both SynCom-amended and unamended plants, maintained at 80% saturation weight by watering with 0.2X MS media. Stress induction began 3 days after SynCom inoculation. The ‘Drought’ condition involved maintaining plants at 40% saturation weight (Gombos et al., 2023) by watering with 0.2X MS media until they were harvested 21 days after stress induction. The ‘Rewatered drought’ condition involved 14 days of drought treatment, followed by restoring saturation weight to 80% for the last week before harvest. The ‘Salinity’ condition involved watering the plants at 80% saturation weight with 0.2X MS media containing 60 mM NaCl. All these plants were incubated in a plant growth chamber (Percival Scientific AR-41 L3, United States) set to 16 h of light, 24°C temperature, and 50% humidity. After 3 weeks of stress induction, plants were harvested by gently removing them from the pots for phenotypic measurements, including shoot wet weight, root length, leaf count, and shoot length (longest leaf). In addition, root tip and root base samples were aseptically harvested as 2 cm cuttings and suspended in 5 mL of 5 mM sodium pyrophosphate + 30 mM phosphate buffer. The sample was sonicated for 10 min and subsequently left on a benchtop for 5 min to allow soil particles to settle down. The supernatant was pipetted out and pelleted to collect microbial cells. DNA extracted from these samples were sent to Novogene, United States, for amplicon sequencing. Quantitative Insights Into Microbial Ecology (QIIME2; Bolyen et al., 2019) was used to process 16S rRNA amplicon data. Within QIIME2, DADA2 was employed for quality filtering, chimera checking, and paired-end read joining. The resulting sequences were trimmed to obtain V4 region (515F-806R), and taxonomic classification was performed using the SILVA database (Quast et al., 2013).

### Assay for plant growth promoting traits in the SynCom strains

Indole-3-Acetic Acid (IAA) assay: The protocol for IAA assay was adapted from Gilbert et al. (2018). Briefly, the isolates were cultured in 5 mL R2A media, with and without 0.5 mg/mL L-tryptophan, for 3 days at 30°C. Following incubation, cultures were centrifuged at 14,000 rpm for 5 min, and 100 μL supernatant was transferred to a 96-well microplate. Subsequently, 200 μL of Salkowski reagent (2% of 0.5 M FeCl_3_ in a 35% HClO_4_ solution) was added to each well. IAA standards (0–10 μg/mL) were also prepared simultaneously. The microplate was incubated in the dark for 30 min, and absorbance values at OD_530_ was recorded to quantify IAA production using the standard curve.

ACC-deaminase activity: The method for measuring 1-Aminocyclopropane-1-carboxylate (ACC) deaminase production in bacterial isolates was adapted from the colorimetric ninhydrin assay described by Li et al. (2011). Briefly, SynCom isolates were cultivated in 2 mL of DF salinity minimum media supplemented with 3 mM ACC for 24 h. Following incubation, 1 mL of the culture was centrifuged at 8,000 x g for 5 min. Subsequently, 100 μL of the resulting supernatant was diluted tenfold, and the ninhydrin assay, with ACC standards ranging from 0.5 mM to 0.005 mM, was used to quantify bacterial ACC consumption.

Phytate solubilization assay: Isolates were inoculated into 3 mL of filter-sterilized phytase-specific medium (PSM; Hosseinkhani and Hosseinkhani, 2009) supplemented with 0.5 mM of Sodium phytate (Fisher Scientific). The cultures were then incubated for 48 h at 30°C in a shaker incubator set at 80 rpm. Following incubation, the OD_600_ was measured, and the cultures were centrifuged at 10,000 rpm for 15 min. The resulting supernatant was retained for a colorimetric assay using the QuantiChrom™ Phosphate Assay Kit (BioAssay Systems, CA, United States). Absorbance was measured at 620 nm and compared against the provided phosphate standards included with the Phosphate Assay Kit.

Siderophore production assay: The capability of siderophore production by the isolates was assessed using the CAS overlaying medium assay (Acharya et al., 2023a; Louden et al., 2011; Pérez-Miranda et al., 2007). Briefly, each isolate was cultured on R2A agar plates at 30°C for 3 days. Subsequently, blue CAS medium with 0.9% agar was overlaid onto the plates. The plates were then further incubated in the same conditions and monitored for color changes over an additional 4 days. The presence of a yellow halo surrounding colonies indicated positive siderophore production. As a positive control, *Burkholderia* sp. PA-E8, a known siderophore producer isolated at our laboratory, was included (Darling et al., 1998; Ong et al., 2016).

Biofilm formation assay: The crystal violet assay for quantifying biofilm formation was adapted from Haney et al. (2021). Briefly, each isolate was grown in R2A media, washed, and resuspended in a 30 mM phosphate buffer to a final OD_600_ of 0.2. The cultures were inoculated into 96-well microtiter plates containing 180 μL of 0.2X MS media, at a 1:10 (v/v) ratio to achieve a final volume of 200 μL (initial OD_600_ of 0.02). The plates were incubated statically at 30°C for 3 days. Post incubation, the liquid fraction was removed by inverting the plates, and each well was washed three times with MilliQ water and air-dried. Subsequently, 100 μL of a 0.1% crystal violet solution (0.1% v/v crystal violet, 1% v/v methanol, and 1% v/v isopropanol in MilliQ water) was added to each well, followed by a 30-min incubation at room temperature. After discarding the staining solution, wells were rinsed three times with MilliQ water. Biofilms were destained with 100 μL of a 30% acetic acid solution and incubated at room temperature for 30 min. OD_595_ of the destaining solution was measured for biofilm quantification.

Gibberellin (GA) assay: The protocol for gibberellin quantification was adapted from Graham and Henderson (1961). Briefly, microbial isolates were cultured in R2A broth at 30°C for 48 h until they reached late-log phase. Cultures were centrifuged at 8,000 rpm for 5 min, and the supernatant was acidified to pH 1.5–2.0 using HCl. An equal volume of ethyl acetate was added, mixed thoroughly, and allowed to separate into organic and aqueous layers. The upper ethyl acetate layer, containing gibberellins, was collected and extracted twice. Combined extracts were vortexed to homogenize, and 50 μL was mixed with 750 μL phosphomolybdic acid reagent in 2 mL tubes. Samples were incubated in a boiling water bath for 1 h, cooled to room temperature, and 200 μL was transferred to a 96-well microplate for absorbance measurement at 780 nm. Gibberellin concentrations were determined using a standard curve prepared from 0 to 10 mg/mL gibberellic acid in absolute alcohol, processed alongside samples.

Acid and Alkaline Phosphatase assay: The protocol for assessing acid and alkaline phosphatase activity was adapted from Sinsabaugh (2001) and Allison (2007). Microbial isolates were cultivated in phytate minimal medium at 30°C until reaching mid-log phase. Cultures were then subjected to OD600 measurements and centrifuged at 8,000 rpm for 5 min. The supernatant was discarded, and the cell pellets were washed three times with, and subsequently resuspended in, 50 mM boric buffer (pH 9) for alkaline phosphatase assays or 50 mM acetate buffer (pH 5) for acid phosphatase assays. For each assay, 750 μL of the cell suspension was mixed with 750 μL of 5 mM p-nitrophenyl phosphate (pNPP) prepared in the corresponding buffer. A substrate control was prepared by combining 750 μL of 5 mM pNPP with 750 μL of buffer alone. All reactions, including isolate samples and controls, were incubated at room temperature for 45 min. Following incubation, samples were centrifuged at 10,000 rpm for 1–2 min. A 750 μL aliquot of the resulting supernatant was transferred to a fresh tube, and 75 μL of 1.0 M NaOH was added to terminate the reaction and facilitate color development. A 300 μL portion of this solution was then transferred to a 96-well microplate, and absorbance was measured at 410 nm. Phosphatase activity was quantified by comparing absorbance values to a standard curve of p-nitrophenol prepared in the corresponding buffer.

Carbon source utilization assay: Each isolate’s growth was assessed on 18 *Brachypodium* root-exudate carbon sources that are significantly enriched during drought stress (Skalska et al., 2021; Fisher et al., 2016; Ahkami et al., 2019; Supplementary Table S2). Cultures were incubated aerobically in 0.2 × MS medium supplemented with 5 mM of each carbon source at 30°C in the dark, with shaking, in biological triplicate. Cell density (OD600) was recorded at 0, 24, 48, 92, 120, 144, and 168 h. The growth was considered positive when the OD600 increased by more than 0.07 relative to the 0-h measurement.

### Statistical analyses

Network analysis and SynCom member interactions: To understand the interspecies interactions from 0.1X R2A enrichments which have the highest diversity and most isolates, the top 50 most abundant ASVs among the core ASVs (present in >75% samples from the 4 generations) from this enrichment were selected. Their abundance matrix across all samples was analyzed using “NetCoMi” package (Peschel et al., 2021) in R. Pearson correlation coefficients greater than 0.3 and Student’s t-test results with p-values less than 0.05 were used to generate a sparse matrix for the network analysis. In the correlation network, each node represents an individual ASV, with different colors representing the corresponding modules. The edges connecting the nodes indicate a strong and significant correlation between the ASVs. The clustering in this network was used to group ASVs into modules that are densely interconnected internally but have sparse connections with other modules.

SynCom strain abundances in the rhizosphere under drought and salinity stress: To identify the changes in individual SynCom member’s abundances under stress, natural log fold differences between the control versus drought, rewatered drought, and salinity conditions were assessed using Analysis of Compositions of Microbiomes with Bias Correction (R package “ANCOMBC”; Lin and Peddada, 2020; Martins et al., 2023). False discovery rates were controlled using the Benjamini-Hochberg method.

Rhizosphere functional redundancy of SynCom members in stress conditions: To test whether the plants under stress modulate rhizosphere microbiome toward similar functions, functional redundancy (FR) across different environments (de Bello et al., 2021) estimated and compared using the R package “SYNCSA” (Debastiani and Pillar, 2012).

## Results

### Selection of isolates to construct a 15-member SynCom

In our previous research, we had developed a number of reduced-complexity microbial enrichments from the rhizosphere of *Brachypodium distachyon* (Chen et al., 2024). We isolated 175 bacterial strains from those enrichments, which we classified by their 16S rRNA genes using the SILVA database (Pruesse et al., 2012). These isolates represented six phyla: Alphaproteobacteria (15), Gammaproteobacteria (124), Actinobacteria (15), Firmicutes (13), Bacteroidetes (7), and Acidobacteria (1).

**Figure 1:**
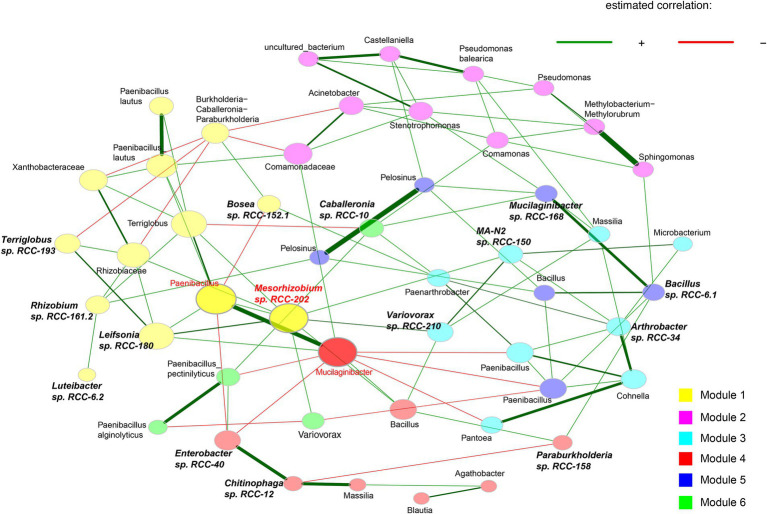
Association networks calculated using the 50 most abundant bacterial ASVs from 0.1X R2A enrichments. The abundance matrices from all samples were loaded into R to construct and visualize the network using the “NetCoMi” package. Networks were generated using the fast greedy clustering algorithm with a Pearson correlation coefficient threshold of ± 0.3, and a t-test (< 0.05) for sparse matrix generation. Eigenvector centrality is used for defining hubs and scaling node sizes. Positive correlations are displayed in green and negative in red. Shape color represents species clusters that are more likely to co-occur with one another than with species outside these modules. Hubs are highlighted in red with corresponding taxon names, whereas SynCom isolates have bolded and italicized names.

**Figure 2:**
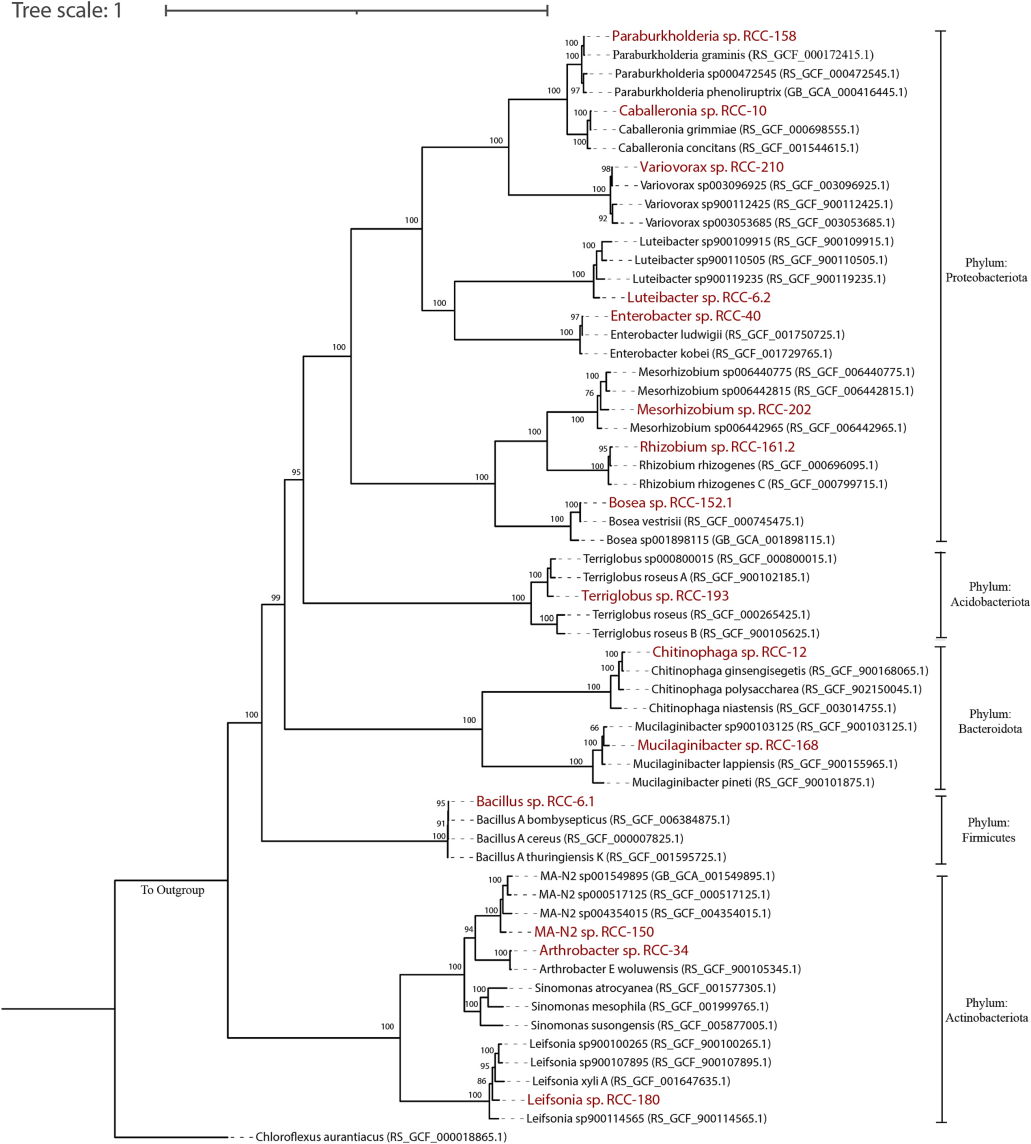
A phylogenomic tree for 15 SynCom isolates was constructed using multiple sequence alignment (MSA) from the concatenation of 120 marker proteins obtained via GTDB-tk workflow. The tree was visualized and edited using iTOL. To create the tree, 2–5 genomes closest to the SynCom isolates were included, with Genbank accession numbers in parentheses. The 15 SynCom members are highlighted in bold red. Phylum-level taxonomy for each genome is indicated on the right side of the tree. Bootstrap values (from 100 replicates) are displayed for nodes over 40 bootstrap supports. *Chloroflexus auranticus* was used as an outgroup to root the tree.

Out of those isolates, we downselected 15 isolates that showed a 100% match in their 16S rRNA gene sequences with both top abundant ASVs from these enrichments (Chen et al., 2024) and with sequences from their rhizosphere origins (Acharya et al., 2023b). To build a SynCom that is truly representative of the *Brachypodium* rhizosphere microbiome, we considered the following criteria: (1) relative abundance of different microbial taxa within the enrichments, (2) representation of phylogenetic diversity among the most abundant microbes, and (3) network interactions among the taxa. Together, the ASVs corresponding to these 15 isolates made up 79.93% of the total reads in the enrichments. We also conducted network analyses on the top 50 ASVs to identify key interactions, which revealed six distinct modules (Figure 1) and a total of 38 positive and 17 negative correlations—24 positive and 9 negative of which occurred among our SynCom members. For the ASVs that matched with selected isolates, six were in Module 1, five in Module 3, three in Module 4, and two each in Modules 5 and 6, which cover most network modules. Some isolates, like *Mesorhizobium* sp. RCC-202 (degree = 8) and *Leifsonia* sp. RCC-180 (degree = 5), showed high network degrees (number of significant correlations connecting to the node), suggesting they play important roles in maintaining network structure. All but one strain, *Bosea* sp. RCC-152.1, had positive connections with at least one other strain across different modules. Informed by all these different analyses, we assembled a SynCom with these 15 isolates that represent taxonomically diverse 5 distinct bacterial phyla (Proteobacteria, Bacteroidota, Acidobacteriota, Actinobacteriota and Firmicutes; Figure 2).

### Genomes of SynCom isolates encode multiple PGP traits

Genomic analysis of nearly complete genomes for all 15 isolates showed that they exhibited a wide range of genome sizes (3.7 Mbp − 8 Mbp), GC content (35–69%), and numbers of genes (3343–7,740). The key general genome features of the SynCom isolates are outlined in Table S1. Most SynCom isolates encoded either a complete set of genes or the essential genes involved in pathways known to be associated with PGP traits (Olanrewaju et al., 2021; Bruto et al., 2014; Gupta et al., 2014; Bhattacharyya and Jha, 2012), potentially contributing to overall plant growth (Table 1). Some of the important PGP traits encoded in the genomes of multiple isolates included 1-aminocyclopropane-1-carboxylate (ACC) deaminase production (9 isolates), Indole-3-acetic acid (IAA) for auxin synthesis (2 isolates), synthesis of antimicrobial compound, i.e., gamma-aminobutyric acid (GABA; 12 isolates), genes associated with plant hormones like amidases (13 isolates), synthesis of volatile organic compounds (VOCs) i.e., 2,3 butanediol and acetoin (14 isolates), trehalose synthesis (13 isolates) siderophore production or transportation (11 isolates), and EPS production (15 isolates; Table 1).

**Table 1:** Plant growth promoting (PGP) traits genes encoded in genomes of SynCom isolates: the table categorizes genes according to their general Plant Growth Promoting Rhizobacteria (PGPR) functions (Column A), specific pathways (Column B), and the presence of these genes in the syncom member genomes (Column C).

Overall function as PGPR	Specific functions	Genes	*Caballeronia* sp. RCC-10	*Chitinophaga* sp. RCC-12	*MA-N2* sp. RCC-150	*Paraburkholderia* sp. RCC-158	*Rhizobium* sp. RCC-161.2	*Mucilaginibacter* sp. RCC-168	*Leifsonia* sp. RCC-180	*Terriglobus* sp. RCC-193	*Mesorhizobium* sp. RCC-202	*Variovorax* sp. RCC-210	*Arthrobacter* sp. RCC-34	*Enterobacter* sp. RCC-40	*Luteibacter* sp. RCC-6.2	*Bosea* sp. RCC-152.1	*Bacillus* sp. RCC-6.1
Plant hormones	Auxin	Auxin/IAA synthseis/ Indole-3-Butyric Acid (IBA is precursor for IAA) *ipdC(4.1.1.74)/ppdC* (indole-3-pyruvate decarboxylase/phenylpyruvate decarboxylase gene)	No	No	No	No	No	No	No	No	No	No	No	Yes	No	No	Yes
	Amidase	Amidase (*amiE*; K01426) [EC:3.5.1.4]	Yes	Yes	Yes	Yes	Yes	Yes	Yes	Yes	Yes	Yes	No	No	Yes	Yes	Yes
	Tryptophan	Tryptophan 2-monooxygenase (*iaaM*; K00466) [EC:1.13.12.3]	No	No	No	Yes	No	No	No	No	No	No	No	No	No	No	No
	Gibberllins	Geranylgeranyl diphosphate synthase (*ggps*) [EC:2.5.1.1]	No	No	Yes	No	No	No	Yes	Yes	No	Yes	Yes	No	No	No	Yes
		Diterpene synthases/cyclases (*cps/ks*) and Cytochrome P450 monooxygenases (cyp112/cyp114/cyp117)	No	No	No	No	No	No	No	No	No	No	No	No	No	No	No
	Putresciene	Ornithine decarboxylase [EC:4.1.1.17] for putresciene synthesis	No	No	No	No	Yes	No	No	No	Yes	No	No	No	No	Yes	No
Stress response	γ-aminobutyric acid (GABA) production	glutamate decarboxylase gadA/B	No	No	Yes	No	No	No	Yes	No	No	No	Yes	No	No	No	Yes
		glutamate: GABA antiporter (gadC)	No	No	No	No	No	No	Yes	No	No	No	No	Yes	No	No	No
		γ-aminobutyric acid (GABA) gene *gabD* K00135 Succinate-semialdehyde dehydrogenase / glutarate-semialdehyde dehydrogenase	Yes	No	Yes	Yes	Yes	No	Yes	Yes	Yes	Yes	Yes	Yes	No	Yes	Yes
	Ubiquinone biosynthesis	ubiB	No	No	No	No	No	No	No	No	No	No	No	No	No	Yes	No
	Leucine catabolism	2-oxoisovalerate dehydrogenase [EC:1.2.4.4]	No	Yes	Yes	No	Yes	Yes	Yes	Yes	Yes	No	Yes	No	Yes	Yes	Yes
	Quorum sensing	*luxS EC 4.4.1.21 and lsrABCD both*	No	No	No	No	No	No	No	No	No	No	No	Yes	No	No	No
	Modulation of plant’s ethylene level by Ethylene synthesis	ACC deaminases (1-aminocyclopropane-1-carboxylate deaminase [EC:3.5.99.7]) Alternative enzyme ([EC:4.4.1.15] dcyD)	Yes	Yes	No	Yes	Yes	Yes	No	No	Yes	Yes	No	Yes	No	Yes	No
	Asparagine utlization	Glutamin-(asparagin-)ase *(aspQ/ansAB)*. 3.5.1.38	No	No	No	No	No	No	No	No	No	Yes	No	No	No	No	No

	H2S production	*cysC K00860*	No	No	No	No	No	No	No	No	Yes	No	No	Yes	No	No	Yes
		*cysJ K00380*	No	No	No	No	No	No	No	Yes	No	No	No	Yes	No	No	No
		*cysI K00381*	Yes	No	No	Yes	Yes	No	No	No	Yes	Yes	No	Yes	No	Yes	No
		*cysN K00956*	Yes	Yes	Yes	Yes	Yes	Yes	No	Yes	Yes	Yes	Yes	Yes	No	Yes	No
	Biofilm formation	lpx genes for for lipid A biosynthesis from KEGG	Yes	Yes	No	Yes	Yes	Yes	No	Yes	Yes	Yes	No	Yes	Yes	Yes	No
		Curli related proteins	Yes	No	No	No	No	No	No	No	No	No	No	Yes	No	No	No
*pgaABCD poly-beta-1-6-N-acetylglucosamine synthesis protein*		No	No	No	No	No	No	No	No	No	Yes	No	Yes	Yes	No	No
Volatile organic compounds (VOCs)	2,3 butanediol and acetoin Synthesis	Acetolactate synthase I/III small subunit (K01653)	Yes	Yes	Yes	Yes	Yes	Yes	Yes	Yes	Yes	Yes	Yes	Yes	Yes	Yes	Yes
		Acetolactate synthase I/II/III large subunit (K01652)	Yes	Yes	Yes	Yes	Yes	Yes	Yes	Yes	Yes	Yes	Yes	Yes	Yes	Yes	Yes
		Threonine 3-dehydrogenase (K00060) [EC:1.1.1.103]	Yes	Yes	Yes	Yes	Yes	Yes	Yes	No	Yes	No	Yes	Yes	Yes	No	Yes
		Acetolactate decarboxylase *budA* (K01575) [EC:4.1.1.5]	No	Yes	No	No	No	Yes	No	No	No	No	No	Yes	No	No	Yes
		(R, R)-butanediol dehydrogenase / meso-butanediol dehydrogenase / diacetyl reductase *budB* (K00004) EC:1.1.1.4 1.1.1.- 1.1.1.303	No	No	No	Yes	No	No	Yes	No	No	No	No	No	No	No	Yes
		Meso-butanediol dehydrogenase / (S, S)-butanediol dehydrogenase / diacetyl reductase *budC* (K18009) EC:1.1.1.304	Yes	No	No	Yes	Yes	No	No	No	No	Yes	Yes	Yes	No	Yes	No
Trehalose	Trehalose Synthesis	Trehalose 6-phosphate phosphatase (o*tsB*; K01087) [EC:3.1.3.12]	Yes	No	Yes	Yes	Yes	No	Yes	No	Yes	Yes	Yes	Yes	Yes	Yes	No
		Trehalose 6-phosphate synthase/phosphatase (o*tsA*; K16055) [EC:2.4.1.15 3.1.3.12]	Yes	Yes	Yes	Yes	Yes	Yes	Yes	No	Yes	Yes	Yes	Yes	Yes	No	No
		Maltooligosyltrehalose trehalohydrolase (t*reZ*; K01236) [EC:3.2.1.141]	Yes	Yes	Yes	Yes	Yes	Yes	Yes	Yes	No	Yes	No	Yes	Yes	Yes	No
		(1- > 4)-alpha-D-glucan 1-alpha-D-glucosylmutase (t*reY*; K06044) [EC:5.4.99.15]	Yes	Yes	Yes	Yes	Yes	Yes	Yes	Yes	No	Yes	No	Yes	Yes	Yes	No
		Maltose alpha-D-glucosyltransferase/ alpha-amylase (*treS*; K05343) [EC:5.4.99.16 3.2.1.1]	Yes	No	Yes	Yes	Yes	No	Yes	Yes	No	Yes	Yes	Yes	Yes	Yes	No
Chemotaxis and motility	Chemotaxis proteins	Chemotaxis protein MotA K02556	Yes	No	No	Yes	Yes	No	Yes	Yes	Yes	Yes	No	Yes	Yes	Yes	Yes
		Chemotaxis protein MotB K02557	Yes	Yes	No	Yes	Yes	Yes	Yes	Yes	Yes	Yes	No	Yes	Yes	Yes	Yes
		Sensor kinase CheA [EC:2.7.13.3]	Yes	No	No	Yes	Yes	No	No	Yes	No	Yes	No	Yes	Yes	Yes	Yes
		Purine-binding chemotaxis protein CheW	Yes	No	No	Yes	Yes	No	No	Yes	No	Yes	No	Yes	Yes	Yes	No
		Chemotaxis protein CheY	Yes	No	No	Yes	Yes	No	No	No	No	Yes	No	Yes	Yes	Yes	No
		Methyl-accepting chemotaxis protein (Mcp) K03406	Yes	No	No	Yes	Yes	No	No	Yes	No	Yes	No	Yes	Yes	Yes	Yes
		chemotaxis protein CheV K03415	Yes	No	No	Yes	No	No	No	No	No	No	No	Yes	Yes	No	Yes
		Protein-glutamate methylesterase/glutaminase [EC:3.1.1.61 3.5.1.44] CheB	Yes	Yes	No	Yes	Yes	Yes	No	Yes	Yes	Yes	No	Yes	Yes	Yes	No
		Chemotaxis protein CheD [EC:3.5.1.44]	Yes	No	No	Yes	Yes	No	No	No	No	Yes	No	No	Yes	Yes	No
		Chemotaxis protein CheC K03410	Yes	No	No	Yes	No	No	No	No	No	No	No	No	No	No	No
		Chemotaxis protein CheZ K03414	Yes	No	No	Yes	No	No	No	No	No	Yes	No	Yes	Yes	No	No
		Chemotaxis protein methyltransferase CheR [EC:2.1.1.80]	Yes	No	No	Yes	Yes	No	No	Yes	No	Yes	No	Yes	Yes	Yes	Yes
	Flagella	Flagellar proteins (fli and flg)	Yes	No	No	Yes	Yes	No	Yes	Yes	Yes	Yes	No	Yes	Yes	Yes	Yes
Mineral phosphate solubilization	Pyrroloquinoline quinone-encoding genes	Quinoprotein glucose dehydrogenase (*gdh*; K00117) [EC:1.1.5.2]	Yes	No	No	Yes	Yes	No	No	No	Yes	No	No	Yes	Yes	No	No
		*pqqB* (pyrroloquinoline quinone biosynthesis protein B)	Yes	No	No	Yes	Yes	No	No	No	No	No	No	No	No	No	No
		*pqqC* [EC:1.3.3.11]	Yes	No	No	Yes	Yes	No	No	No	No	No	No	No	No	No	No
		pyrroloquinoline quinone biosynthesis protein D *pqqD*	Yes	No	No	Yes	Yes	No	No	No	No	No	No	No	No	No	No
		*pqqE* (PqqA peptide cyclase [EC:1.21.98.4])*, pqqF, pqqG, pqqA*	No	No	No	No	No	No	No	No	No	No	No	No	No	No	No
		All three genes *pstABC* (phosphate transport system). K02038, K02036, K02037	Yes	No	Yes	Yes	Yes	No	Yes	Yes	Yes	Yes	Yes	Yes	Yes	Yes	Yes
Siderophore to scavenge iron and other essential elements	Siderophore production	*entB (K01252) [EC:3.3.2.1 6.3.2.14]* *entA (K00216) [EC:1.3.1.28]* *entD (K02362) [EC:6.3.2.14]* *entF (K02364) [EC:6.3.2.14 6.2.1.72]* *entE (K02363)[EC:6.3.2.14 6.2.1.71]* *entC isochorismate synthase (K02361) [EC:5.4.4.2]*	No	No	No	No	No	No	No	No	No	No	No	Yes	No	No	Yes
	Siderophore transportation	siderophore transport protein TonB	Yes	Yes	No	Yes	Yes	Yes	No	Yes	Yes	Yes	No	Yes	Yes	Yes	No
		siderophore transport protein ExbD	Yes	Yes	No	Yes	No	Yes	No	Yes	Yes	Yes	No	Yes	Yes	Yes	No
		siderophore transport protein ExbB	Yes	Yes	No	Yes	No	Yes	No	Yes	Yes	Yes	No	Yes	Yes	Yes	No
Organic phosphorus mineralization	Phytate mineralization	Alkaline phosphatase D (*phoD*; K01113) [EC:3.1.3.1]	Yes	Yes	No	No	No	Yes	Yes	No	Yes	Yes	No	Yes	No	No	No
		Myo-inositol-hexakisphosphate 4-phosphohydrolase, 4-phytase (EC 3.1.3.26)	No	No	No	No	No	No	No	No	No	No	No	No	Yes	No	No
		Protein tyrosine phosphatase [EC:3.1.3.48]	Yes	Yes	Yes	Yes	No	Yes	Yes	No	Yes	Yes	Yes	Yes	Yes	No	Yes
Exopolysaccharide (EPS) synthesis	Rhizobial exopolysaccharide (EPS)	Exopolysaccharide production protein ExoY (K16566)	No	No	Yes	No	Yes	No	Yes	Yes	Yes	Yes	Yes	No	No	Yes	No

Succinoglycan biosynthesis protein ExoA (K16557)		No	No	No	No	Yes	No	Yes	No	Yes	No	No	No	No	No	No

Exopolysaccharide production protein ExoZ (K16568)		Yes	Yes	Yes	Yes	Yes	Yes	Yes	Yes	Yes	Yes	Yes	Yes	Yes	Yes	Yes

Succinoglycan biosynthesis protein ExoO (K16555), ExoU (K16564), ExoM (K16556), ExoL (K16558)	No	No	No	No	Yes	No	No	No	Yes	No	No	No	No	No	No

Succinoglycan biosynthesis protein ExoW (K16562), ExoH (K16560), ExoV (K16563)	No	No	No	No	Yes	No	No	No	No	No	No	No	No	No	No

Succinoglycan biosynthesis protein ExoQ	No	No	No	No	Yes	No	Yes	No	Yes	Yes	No	No	No	No	No

Vibrio polysachharide	Serine O-acetyltransferase [EC:2.3.1.30], cysE (K00640)	Yes	Yes	Yes	Yes	Yes	Yes	Yes	Yes	Yes	Yes	Yes	Yes	Yes	Yes	Yes

Enterobacterial commom antigen pathway	UDP-GlcNAc:undecaprenyl-phosphate [EC:2.7.8.33], WecA (K02851)	No	Yes	Yes	No	No	Yes	Yes	No	No	No	No	Yes	No	No	Yes

	UDP-N-acetyl-D-mannosaminouronate [EC:2.4.1.180], WecG (K02852)	No	No	No	No	No	No	No	No	No	No	No	Yes	No	No	No

dTDP-N-acetylfucosamine [EC:2.4.1.325], WecF (K12582)	No	No	No	No	No	No	Yes	No	No	No	No	Yes	No	No	No

Psl polysaccharide	Polysaccharide biosynthesis protein PslA (K20997)	No	No	No	No	Yes	No	No	No	Yes	No	No	No	No	No	No

	Polysaccharide biosynthesis protein PslC (K25205), PslI (K21002)	No	No	No	No	No	No	No	No	No	No	No	No	No	No	No

Polysaccharide biosynthesis protein PslF (K20999)	No	Yes	No	No	No	Yes	No	No	No	No	No	No	No	No	No

Polysaccharide biosynthesis protein PslH (K21001)	No	Yes	No	No	Yes	Yes	No	No	Yes	No	No	No	Yes	No	No

Poly-N-acetyl-glucosamine	Poly-beta-1,6-N-acetyl-D-glucosamine synthase, pgaC, icaA (K11936)	No	Yes	Yes	No	No	Yes	No	Yes	No	Yes	No	Yes	Yes	No	No

Biofilm PGA synthesis protein PgaD (K11937)	No	No	No	No	No	No	No	No	No	No	No	Yes	Yes	No	No

Colanic acid synthesis (Extracellular/LPS type)	Colanic acid biosynthesis glycosyltransferase WcaI (K03208)	Yes	Yes	No	Yes	No	Yes	No	Yes	No	Yes	Yes	No	No	No	No

	Colanic acid biosynthesis acetyltransferase WcaF (K03818)	Yes	Yes	No	Yes	No	Yes	No	Yes	No	No	No	Yes	No	No	No

	Colanic acid biosynthesis glycosyltransferase WcaE (K13683)	No	Yes	No	No	No	Yes	No	No	No	No	No	Yes	No	No	No

Colanic acid biosynthesis glycosyltransferase WcaC (K13684), WcaB (K03819), WcaA (K25875) Undecaprenyl-phosphate glucose phosphotransferase WcaJ (K03606)	No	No	No	No	No	No	No	No	No	No	No	Yes	No	No	No

Colanic acid biosynthesis glycosyltransferase WcaL (K16703)	No	No	No	No	Yes	No	No	No	No	No	No	Yes	Yes	No	No

Colanic acid biosynthesis protein WcaK (K16710)	No	No	No	No	No	No	No	No	No	No	No	Yes	Yes	No	No

EPS regulator genes for *Pseudomonas putida*	surface adhesion protein lapA (K12549)	No	No	No	No	No	No	No	No	No	Yes	No	No	No	No	No

	Cellulose biosynthesis protein BcsQ (PF06564.15)	Yes	No	No	Yes	Yes	No	No	Yes	No	Yes	No	Yes	No	Yes	No

alginate O-acetyltransferase complex protein AlgI (K19294)	No	Yes	No	No	No	Yes	No	Yes	No	Yes	No	No	Yes	Yes	No

Genes found in the SynCom genomes are highlighted in green, while those that are absent are marked in red.

### SynCom strains express multiple PGP traits during phenotypic characterization

We tested eight PGP traits essential for plant’s growth by conducting phenotypic assays. These include IAA production, ACC deaminase activity, phytate solubilization, siderophore production, biofilm formation, Gibberellin production, and Acid and Alkaline Phosphatase assay (Table 2). Six SynCom isolates produced IAA in the presence of L-tryptophan, with concentrations ranging from 0.25 to 23 μg/mL. The highest IAA levels (~23 μg/mL) were observed in *Enterobacter* sp. RCC-40 and *Mucilaginibacter* sp. RCC-168. All SynCom isolates exhibited ACC deaminase activity, with nine strains degrading more than 50% of the supplied 3 mM ACC. Siderophore production was detected in seven isolates, indicated by the formation of a yellow halo in the o-CAS assay (Table 2). Ability to form biofilms, as assessed by crystal violet assay, was significant (*p*-value < 0.05) by Student’s t-test in nine isolates. All the isolates produced Gibberellins, with Bacillus sp. RCC-6.1 and Enterobacter sp. RCC-40 showing the highest levels. Five isolates, including *Luteibacter* sp. RCC-6.2, *Terriglobus* sp. RCC-193, *Mucilaginibacter* sp. RCC-168, *Leifsonia* sp. RCC-180, and *Rhizobium* sp. RCC-161.2, demonstrated phytate solubilization with phosphorus release in the range of 0.0003–0.006 mg/dL. Six isolates produced acid phosphatase, with Bosea sp. RCC-152.1 and Enterobacter sp. RCC-40 exhibited the highest activity (~600 uM), while the remaining isolates produced <200 uM. Lastly, five isolates produced alkaline phosphatase, with Enterobacter sp. RCC-40 and Arthrobacter sp. RCC-34 displaying the highest concentrations (146–375 uM; Table 2). Notably, *Mucilaginibacter* sp. RCC-168 tested positive for all eight PGP traits. We also evaluated each strain’s growth on 18 drought-enriched *Brachypodium* exudates (Skalska et al., 2021; Fisher et al., 2016; Ahkami et al., 2019). Eleven of these compounds were metabolized by every isolate (Supplementary Figure S1). *Paraburkholderia* sp. RCC-158 grew on all 18 substrates and exhibited the most rapid growth as indicated by OD600 increase (Supplementary Figure S2).

**Table 2:** Results for the plant growth-promoting (PGP) traits assays in SynCom strains phenotypic characterization.

SynCom isolates	IAA production (ug/ml)	ACC consumed (mM)	Phytate Solubilization (mg/dL)	Siderophore Production	Biofilm formation (OD595)	Gibberlin production (ug/ml)	Acid phosphatase concentration (uM)	Alkaline Phosphatase concentration (uM)
*Bosea* sp. RCC-152.1		2.25		**+**	0.282	74.614	616.554	
*Luteibacter* sp. RCC-6.2		2.03	0.0003		0.337	38.411		
*Bacillus* sp. RCC-6.1		2.01		**+**	0.269	101.545		
*Enterobacter* sp. RCC-40	23.32	1.97		**+**	0.898	107.726	566.388	375.879
*Paraburkholderia* sp. RCC-158		1.75		**+**		65.784	196.436	58.133
*Caballeronia* sp. RCC-10		1.72			0.359	56.512		
*Arthrobacter* sp. RCC-34	8.30	1.64			0.736	68.874	15.036	146.804
*Variovorax* sp. RCC-210		1.61			0.469	32.671		
*MA-N2* sp. RCC-150		1.57				52.539		
*Terriglobus* sp. RCC-193	8.83	1.56	0.0026			65.783		
*Chitinophaga* sp. RCC-12		1.40		**+**		2.208	151.573	
*Mesorhizobium* sp. RCC-202	0.25	1.39			0.259	28.256		
*Mucilaginibacter* sp. RCC-168	23.32	1.34	0.0043	**+**	0.316	30.464	47.598	1.124
*Leifsonia* sp. RCC-180		1.31	0.0065			51.656		
*Rhizobium* sp. RCC-161.2	3.13	1.17	0.0026	**+**		58.720		11.394

Results summarizing the phenotypic assays for the plant growth-promoting (PGP) traits in SynCom isolates. For each column/tests darker green represents higher values and lighter color represents smaller values within the column whereas no values represents negative results. 1) IAA production: Amount of IAA produced by the individual isolates when cultured in media containing 0.5mg/mL L-tryptophan and subjected to the Salkowski calorimetric test. 2) ACC deaminase production: Values here indicate the quantity of ACC degraded (in mM) by the individual isolates when cultivated in media supplemented with 3 mM ACC. Higher number represents higher levels of ACC deaminase production. 3) Phytate Solubilization: The values presented here signify the amount of phytate consumed (in mg/dL) by each bacterium during their growth in phytate-specific media containing 0.5 mM Sodium phytate. 4) Siderophore Production: (+) denotes positive siderophore production by the SynCom isolates characterized by the formation of a halo surrounding bacterial cultures upon overlaying with a blue-colored solution after 4 days of growth in a qualitative plate assay. 5) Biofilm formation: Average OD595 values of the wash solution at the end of the biofilm assay of SynCom strains in MS media. The values for OD for the strains that had a significantly higher OD than the negative control (0.216) are listed here. 6) Gibberlin production: Amount of Gibberlin produced by the individual isolates as indicated by the reaction of Gibberlins with Phosphomolybdic acid reagent. 7) Acid phosphatase assay: The numbers represents the average standardized concentration of p-nitrophenol (uM) in Acetate Buffer (pH = 5) representing acid phosphatase activity in the isolates. 8) Alkaline phosphatase assay: The numbers represents the average standardized concentration p-nitrophenol (uM) in Boric Buffer (pH = 9) representing alkaline phosphatase activity in the isolates.

### SynCom strains exhibit better persistence in planta

**Figure 3:**
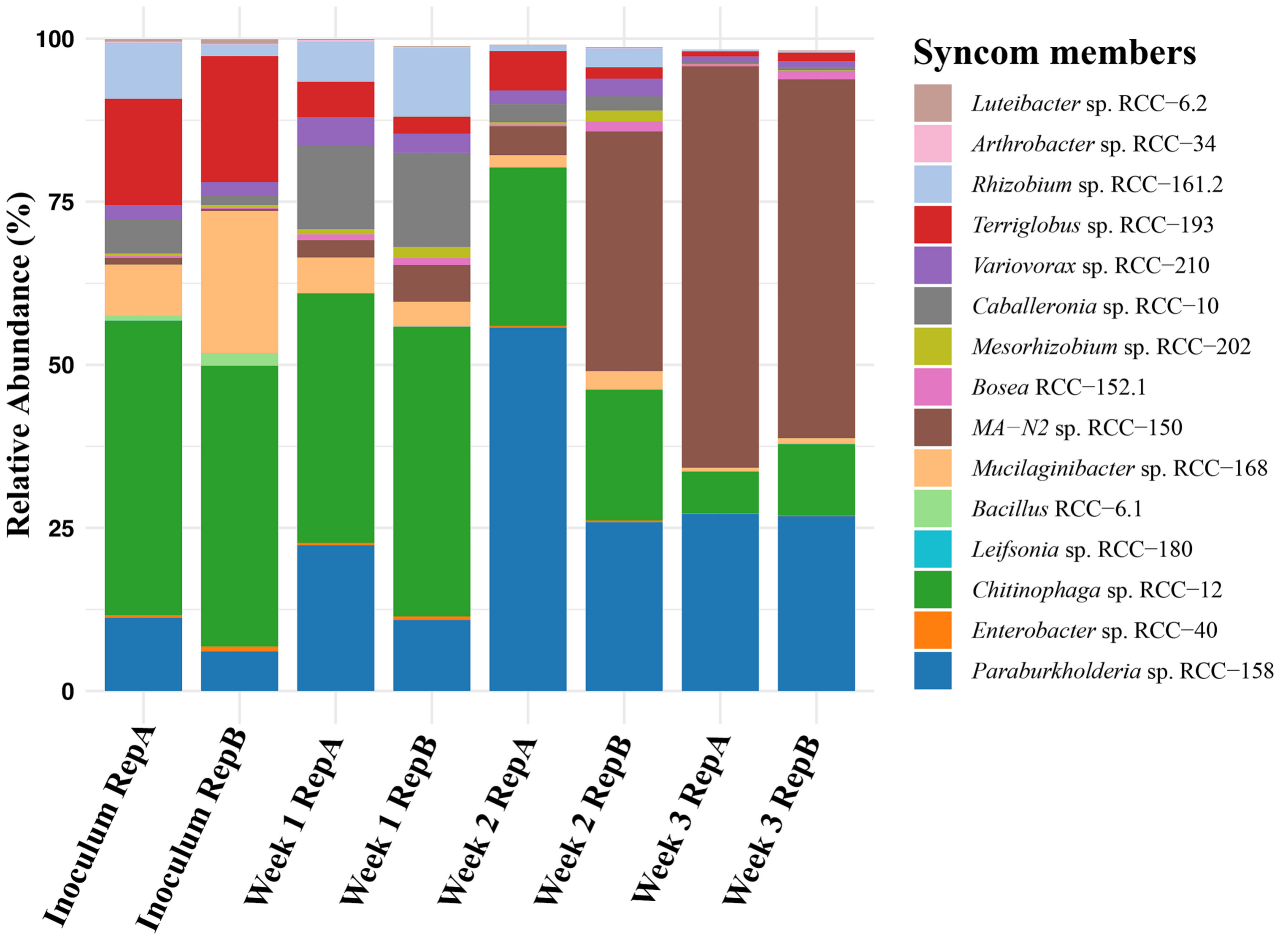
Stability of Syncom members: Changes in the microbial community over 3 weeks when the 15-member SynCom is cultured in 0.2X MS media and transferred weekly. The initial bar on the x-axis reflects the relative abundance of 15 SynCom members in the Inoculum, followed by pairs of bars representing duplicates for samples taken at the end of Weeks 1, 2, and 3. The Y-axis shows the relative abundances of each SynCom strain which is represented by a different color as shown in the legend.

Since all strains were isolated from the plant rhizosphere, we hypothesized that the presence of the plant host would positively impact SynCom growth and stability. To test this, we first examined SynCom growth by combining 15 strains in equal cell volumes and inoculating them into 0.2X MS media, which we generally used to water the plants. Samples were collected weekly, and community composition assessed through 16S rRNA (V3-V4 region) sequencing (Figure 3). The results showed that six of the 15 strains were undetectable or had a relative abundance below 0.1% after 3 weeks. Notably, the *in vitro* environment did not support the growth of Gram-positive strains, as three (*Arthrobacter* sp. RCC-34, *Leifsonia* sp. RCC-180, and *Bacillus* sp. RCC-6.1) of the four were either absent or present at very low abundance (<0.1%) when sampled. Conversely, members such as *MA-N2* sp. RCC-150, *Paraburkholderia* sp. RCC-158 and *Chitinophaga* sp. RCC-12, dominated the culture at the end of week 3 with an average relative abundance of 60, 26 and 8%, respectively (Figure 3).

**Figure 4:**
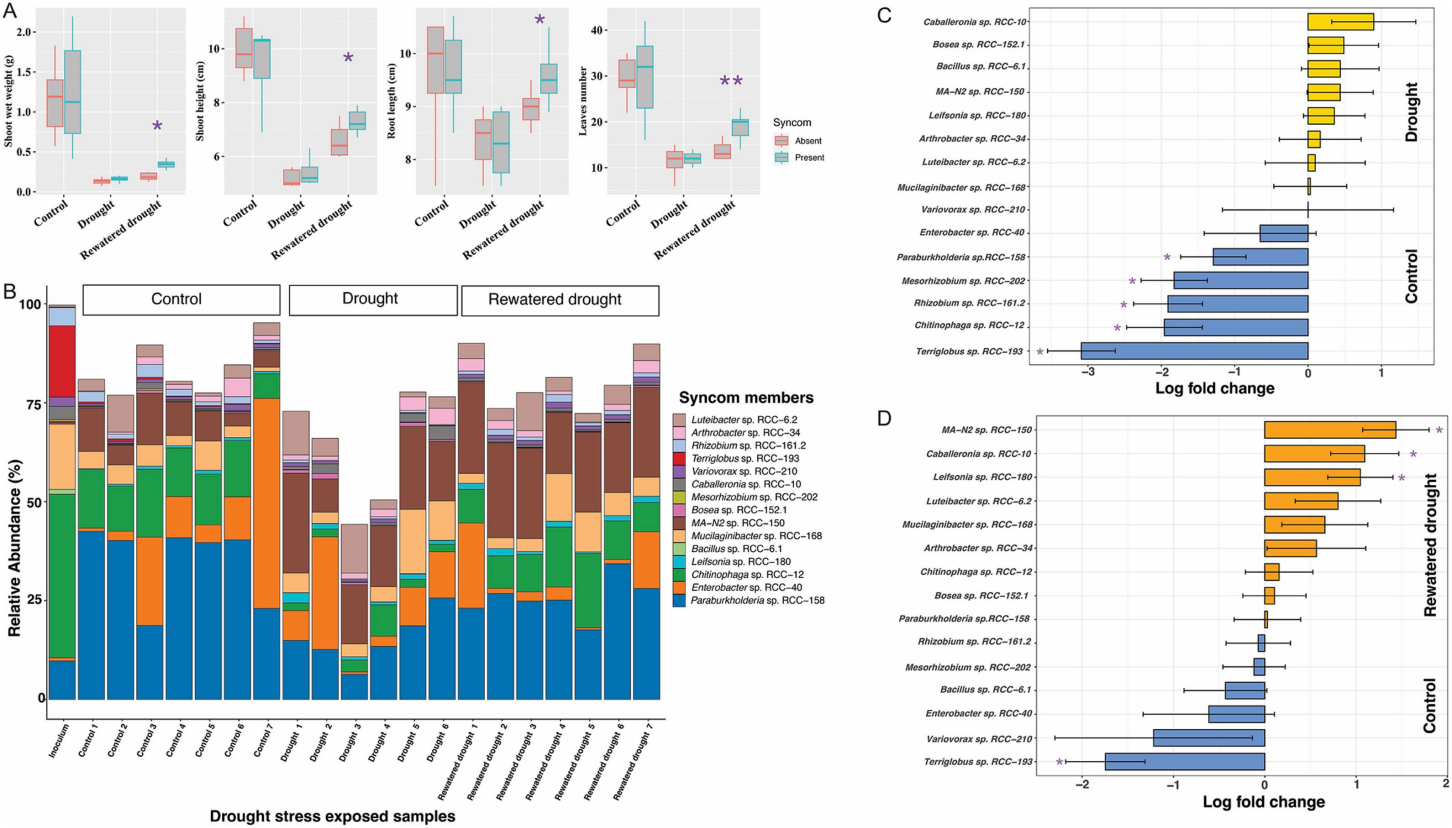
Figures depicting the plant phenotypic traits and microbial 16S rRNA gene abundances profile from the SynCom drought stress experiment. The experiment comprised three sets: the Control set, Drought set, and Rewatered Drought set. **(A)** Plant phenotype results: Comparisons for the shoot weight, shoot height, root length, and leaves numbers in *Brachypodium*, with and without SynCom members, during a 3-week exposure to drought stress. Each experimental category included seven *Brachypodium* plants. Significant values determined by t-tests are indicated by purple asterisks positioned above the corresponding bars. **(B)** 16S rRNA gene abundances profiles: In the barplot, each bar represents a replicate (shown in x-axis) in the corresponding experimental set as indicated above the bars. Y-axis shows the relative abundances values for each SynCom isolates represented by different color in legend, while all other bacterial groups are combined into the “Others” group, depicted in white on the plot. It is important to note that certain DNA samples in the experimental sets, i.e., rewatered drought encountered sequencing issues, leading to a reduced sample size of 5. **(C)** The natural log fold changes (lfc) for SynCom strains in stress conditions, as measured by “ANCOMBC” with drought (yellow) and **(D)** rewatered drought (orange), when compared to the SynCom-amended control plants (blue). On y-axis, negative values indicate that taxa are more abundant in control conditions, while positive values show greater abundance in drought or rewatered drought conditions. Bars indicate changes in the natural log fold, and error bars represent standard errors. Asterisks indicate significant changes in the natural log fold after the Benjamini-Hochberg adjustment of the false discovery rate (*p* < 0.05).

Next, we evaluated the growth and persistence of SynCom members in the presence of *Brachypodium*. After 3 weeks of growth *in planta*, the relative abundance of strains was more evenly distributed (Figure 4B). Notably, Gram-positive strains such as *Arthrobacter* sp. RCC-34 (1.4%) and *Leifsonia* sp. RCC-180 (0.6%) showed substantial increases in relative abundance compared to *in vitro* conditions. In contrast, *Bacillus* sp. RCC-6.1, showed a low relative abundance (<0.1%). Additionally, 11 of the 15 strains displayed higher median relative abundances when grown with the plant than without (Supplementary Figure S3).

### Drought and salinity stress in *Brachypodium* enrich specific SynCom strains

**Figure 5:**
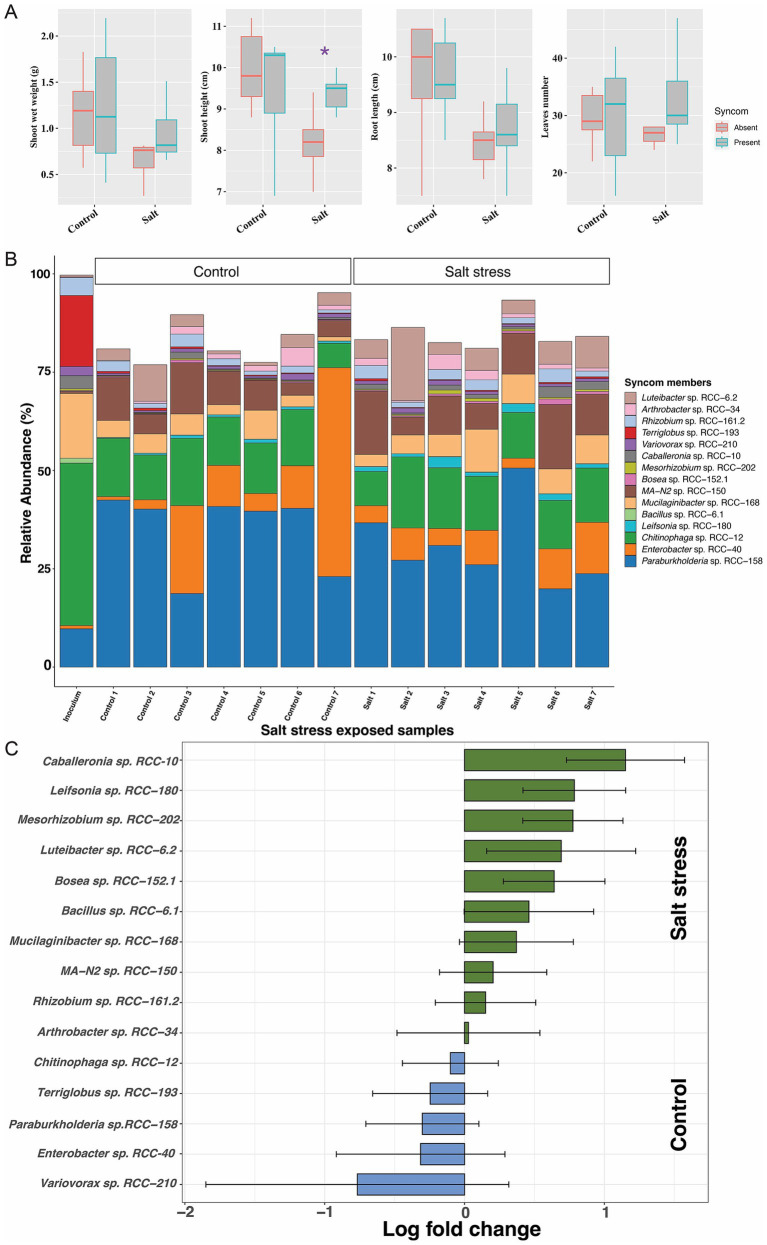
Figures depicting the plant phenotypic traits microbial 16S rRNA gene abundances profile from the SynCom salt stress experiment. The experiment comprised two sets, i.e., the Control set and the Salt stress. **(A)** Plant phenotype result: Comparisons for the shoot weight, shoot height, root length and leaves numbers in *Brachypodium*, with and without SynCom members, during a 3-week exposure to salt stress. Each experimental category included seven *Brachypodium* plants. Significant values determined by t-tests are indicated by purple asterisks positioned above the corresponding bars. **(B)** 16S rRNA gene abundance profiles: In the barplot, each bar represents a replicate (shown in x-axis) in the corresponding experimental set as indicated above the bars. Y-axis shows the relative abundances values for each Syncom isolate represented by different color in the legend, while all other bacterial groups are combined into the “Others” group, depicted in white on the plot. **(C)** The natural log fold changes (lfc) for SynCom strains in stress conditions, as measured by “ANCOMBC” with salt stress (green) when compared to the SynCom-amended control plants (blue). On y-axis, negative values indicate that taxa are more abundant in control conditions, while positive values show greater abundance in salt conditions. Bars indicate changes in the natural log fold, and error bars represent standard errors. None of the SynCom members was found to have significant changes in natural log fold after the Benjamini-Hochberg adjustment of false discovery rate (*p* < 0.05).

We assessed the impact of the SynCom on plant phenotype under drought and salinity stress, two common environmental stressors that plants encounter. No significant differences were observed between SynCom-amended and unamended plants with regards to shoot height, shoot weight, root length, or leaf number under drought stress (Figure 4A; Supplementary Figure S7). However, SynCom-amended plants showed significant improvements across all these above measured parameters (t-test, Figure 4A) on rewatering after drought. Under salinity stress, SynCom-amended plants exhibited a significant increase in shoot height compared to unamended plants (Figure 5A).

When examining SynCom persistence under the above stress conditions, we observed that all strains were persistent throughout drought, rewatered drought, and salinity stress after 3 weeks (Figures 4B, 5B). However, significant differences in relative abundance of the individual strains were observed. Under drought, *Paraburkholderia* sp. RCC-158 (lfc = 1.29), *Rhizobium* sp. RCC-161.2 (lfc = 1.91), *Mesorhizobium* sp. RCC-202 (lfc = 1.82), *Terriglobus* sp. RCC-193 (lfc = 3.09), and *Chitinophaga* sp. RCC-12 (lfc = 1.96) showed significant (*p* < 0.05, two-sided Chi-square test) decreases in log fold change (lfc) relative abundance (Figure 4C) compared to the no-stress control. In rewatered drought conditions, *Terriglobus* sp. RCC-193 (lfc = 1.74) showed a significant decrease, while *MA-N2* sp. RCC-150 (lfc = 1.4), *Leifsonia* sp. RCC-180 (lfc = 1.04), and *Caballeronia* sp. RCC-10 (lfc = 1.09) significantly increased (Figure 4D) relative to controls. No significant change in SynCom relative abundance was observed under salinity stress (Figure 5C).

### SynCom strains exhibit distinct spatial localization on the root

**Figure 6:**
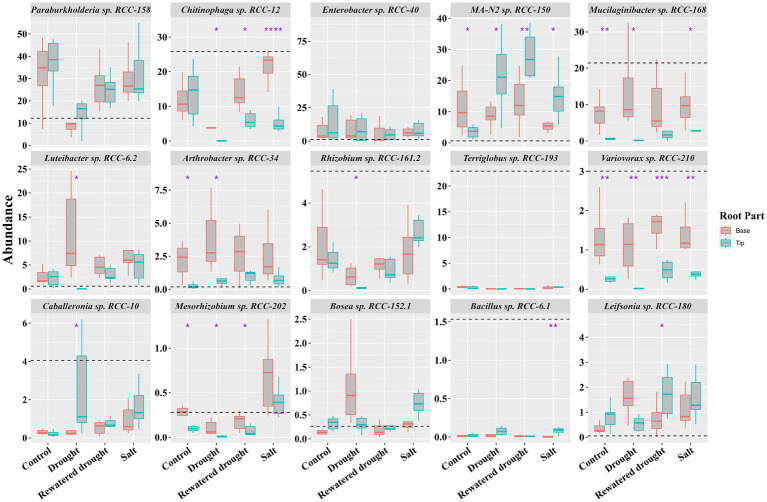
Spatial localization of SynCom strains in the Brachypodium root system: Boxplots show the 16S rRNA gene abundances for the SynCom isolates across the root tip and base in SynCom-amended plants under control and drought and salt stress conditions (as indicated on the x-axis) after 21 days. The y-axis displays the relative percentage abundances of the SynCom members indicated atop each boxplot panel. Significant differences determined by t-tests between the root parts (tip and base) within each condition are marked with an asterisk above and between the respective bars. The black horizontal dashed line indicates the percentage of strain abundance in the inoculum.

Previous studies have highlighted that plants secrete specific compounds as root exudates to recruit select microbes, especially under stressful conditions (Vives-Peris et al., 2020). Given that exudation might vary between root tips and base (Dragišić Maksimović et al., 2021), we investigated the recruitment and spatial localization of SynCom strains on different parts of the root in the experiments above (drought and salinity stress). The SynCom was applied to the seedling’s growing root base for both these tests, and at the completion of the experiments, we analyzed the community at both the root tip and root base using 16S rRNA-based amplicon sequencing. Distinct colonization patterns emerged for certain strains. Under no stress, strains such as *MA-N2* sp. RCC-150, *Mucilaginibacter* sp. RCC-168, Var*iovorax* sp. RCC-210, and *Arthrobacter* sp. RCC-34 were significantly enriched (t-test) at the root base compared to their abundance in the inoculum and root tips (Figure 6, control sets). Under drought stress, however, *Luteibacter* sp. RCC-6.2 and *Arthrobacter* sp. RCC-34 showed increased abundance at the base, while *MA-N2* sp. RCC-150 was significantly enriched at the root tip. In rewatered drought conditions, *MA-N2* sp. RCC-150 and *Leifsonia* sp. RCC-180 were more abundant at the root tip. During salinity stress, *MA-N2* sp. RCC-150 was the only strain found to have significantly increased abundance at the root tip. Overall, *MA-N2* sp. RCC-150 displayed a notable preference for the root tip, especially under salt and drought stress conditions.

## Discussion

We developed a framework to integrate both culture-dependent and culture-independent approaches toward creating a stable SynCom that interacts positively with the host plant and confers beneficial traits during adverse environmental conditions. To do this, several strains were isolated from a *Brachypodium* rhizosphere enriched community, and selection of a subset was designed informed by 16S rDNA amplicon sequencing of enriched communities, and network analysis, which revealed both positive and negative microbial interactions within the enriched community (Chen et al., 2024; Figure 1). These interactions informed the final selection of microbial members of the SynCom, ensuring ecological relevance and functional integrity (Toju et al., 2020). At the end of our data-driven SynCom assembly, the final 15 strains included representation from five different phyla (Figure 2). Notably, *Mesorhizobium* sp. RCC-202, a keystone species identified in the network analysis (Figure 1), was included for its critical role in shaping microbial community structure and dynamics that likely contributes to the SynCom’s robustness (Agler et al., 2016). We were also successful in including *Terriglobus* (*Terriglobus* sp. RCC-193), a genus within Acidobacteria known for its ecological significance in soil and its challenging cultivation, highlighting our effort to incorporate underexplored yet essential microbial taxa (Eichorst et al., 2007; Kalam et al., 2022). A persistent challenge in SynCom development for enhancing plant productivity is identifying which specific microbial members actually contribute to community stability and functional outcomes (Pradhan et al., 2022; Sun et al., 2025; O’Banion et al., 2020). Here, we were successful in assembling a Syncom with the keystone species based on network interaction (Figure 1) and fairly persistent microbial culture propagated *in vitro* (Figure 3). In subsequent experiments, we show that all SynCom members demonstrated persistence in the *Brachypodium* rhizosphere over a period of 3 weeks, even under stress conditions (Figures 4, 5). Notably, their improved persistence and more balanced relative abundance *in planta* compared to *in vitro,* suggest positive interactions with the host plant, an often-overlooked criterion in previous SynCom studies (Sanchez-Gorostiaga et al., 2018; Toju et al., 2020; Werner and Kiers, 2015; Kabir et al., 2024). Additionally, the sustained presence of Gram-positive bacteria, in particular, is noteworthy due to their role and potential for use as microbial amendments in agricultural applications for enhancing host plant resilience during environmental stress (Schimel et al., 2007; Qi et al., 2022).

We examined specific colonization of the SynCom members along the root of *Brachypodium distachyon* (Figure 6). It is generally accepted that the spatial distribution of microbes along the root surface is in direct response to differential root exudation and gradients thereof (Wei et al., 2021; Acharya et al., 2023b). We observed a strong preference for colonization at the root tip under stress conditions (Figure 6), which demonstrates that the microbes migrated to the root tip with the growing root despite being inoculated initially to the root base. This was particularly evident for Gram-positive strains such as *MA-N2* sp. RCC-150, *Leifsonia* sp. RCC-180, and *Bacillus* sp. RCC-6.1 (Figure 6). Since the root tip is the most actively growing part of the root, this result indicates that when growing under stress, the plant is actively seeking to recruit specific microbes that help with stress alleviation. It is known that *Brachypodium* increases production of levels of metabolites like proline and soluble sugars in drought-tolerant varieties (Shi et al., 2015). These compounds can serve as chemoattractant and carbon sources for Gram-positive bacteria (Gagné-Bourque et al., 2015; Sharma et al., 2020). Therefore it is reasonable to assume that Gram-positive bacteria, such as Actinobacteria and Bacilli, produce metabolites that assist in drought stress tolerance are attracted to the root tip through these root exudate compounds (Ek-Ramos et al., 2019; Grover et al., 2016). Our carbon-utilization phenotypic assays (Supplementary Figure S1) revealed that the Gram-positive taxa—*MA-N2* sp. RCC-150, *Leifsonia* sp. RCC-180, and *Arthrobacter* sp. RCC-34—each metabolized large subsets of metabolites that significantly enriched during the drought stress (e.g., proline, malate, sucrose). Notably, *MA-N2* sp. RCC-150 and *Arthrobacter* sp. RCC-34 were among only three isolates able to utilize malate, a key drought-linked carbon source that increased significantly in both roots and leaves for *Brachypodium* (Ahkami et al., 2019). This in vitro preference for osmoprotectants and sugars parallels their in planta enrichment at the growing root tip, underscoring their role in exploiting exudates under stress. Specifically, *MA-N2* sp. RCC-150 showed increased overall abundance during rewatered drought conditions (Figure 4) and was enriched at the root tips during both drought and rewatered drought (Figure 6). The root tip is often considered to be the part of the root of active root exudation. Therefore this enrichment is likely due to multiple factors, including the strain’s ability to respond to specific root exudate metabolites (Supplementary Figure S1) or, ability to return to the plant trehalose and various exopolysaccharides that potentially benefit the plant during drought stress (Table 1).

When we compared growth of *Brachypodium* in SynCom-amended and unamended treatments under different stress conditions, we found that the SynCom exhibited substantially higher functional redundancy under drought and rewatered drought conditions compared to no-stress conditions (Supplementary Figure S5). This suggests that bacterial strains enriched during stress encode overlapping PGP traits (Table 1), with functional redundancy likely acting as a buffer to maintain critical microbial functions despite shifts in community composition. These findings align with evidence that plants under stress modulate their rhizosphere microbiomes based on shared PGP functions rather than recruiting specific taxa (Louca et al., 2018; Zia et al., 2021). Notably, while significant changes in SynCom composition occurred during both drought and rewatered drought treatments, measurable enhancements in plant phenotypes, such as above- and below-ground biomass, and root and shoot heights, were observed only in rewatered drought treatments (Figure 4A). These observations align with emerging research suggesting microbial effects on plant recovery post-stress can be more pronounced than during the stress itself (Bandopadhyay et al., 2024). It is known that many bacteria enter dormant or low-metabolism states to conserve energy during drought stress (de Vries et al., 2020). Upon rehydration, microbial communities can rapidly rebound, with increased metabolic activity from the host plants including flush of root exudates (Canarini et al., 2019), colonization, and production of plant growth-promoting compounds such as auxins, gibberellins, and ACC deaminase (Rolli et al., 2015; Ngumbi and Kloepper, 2016). Furthermore, microbial consortia may “prime” plants during drought for enhanced resilience, with effects becoming measurable only once stress is relieved and growth resumes (Santos-Medellín et al., 2017).

Recognizing that microbial effects on plants are often more pronounced during post-stress recovery than during the stress itself, the notable benefits of SynCom to host plants during the rewatered drought conditions can be attributed to several factors. First, the isolates with significantly increased relative abundance under rewatered drought (e.g., *MA-N2* sp. RCC-150, *Caballeronia* sp. RCC-10, and *Leifsonia* sp. RCC-180; Figure 4D), belong to genera known to alleviate drought stress in plants (Nordstedt et al., 2021; Platamone et al., 2023), and their genomes encode genes for producing osmoprotectant compounds (Supplementary Table S2) and exopolysaccharide production protein (Table 1). Second, several other SynCom strains harbor genes critical for managing drought and salinity stresses, including genes for trehalose biosynthesis (Kosar et al., 2018; Sharma et al., 2020; Hassan et al., 2023), sodium and potassium transporters (Ahmad et al., 2022; Gunde-Cimerman et al., 2018), and ACC deaminase (Orozco-Mosqueda et al., 2020; Table 1). Third, we observed enhanced positive interactions among SynCom strains, particularly during rewatered drought (22 positive and 7 negative, Supplementary Figure S6). These positive microbial interactions likely promoted overall microbial community functionality and benefited the host plant (Layeghifard et al., 2017; Martins et al., 2023). Our findings overall demonstrate that SynCom persists under drought conditions and supports more effective plant recovery post-drought, emphasizing their potential role for applications in enhancing crop resilience and productivity amid climate variability. While we can postulate that certain genes and traits of the SynCom strains play a role in alleviating drought, future experiments exploring gene-expression and metabolite production are needed to confirm.

To summarize, in this study, we developed a 15-member SynCom from the rhizobiome of *Brachypodium distachyon* using an integrative framework that combined culture-dependent and culture-independent methods, guided by network analysis to ensure ecological relevance and functional integrity. The SynCom demonstrated persistence, stability when grown with plants, and conferred resilience under stress conditions, aiding plant recovery during rewatering after drought. These findings highlight the SynCom’s potential for agricultural applications in improving crop resilience and supporting recovery after drought or salinity stress. Future research exploring SynCom influence on plant gene expression and vice versa under drought and salt stress will further help optimize the use and application of this SynCom in diverse agricultural systems, ensuring scalability and efficacy in mitigating climate-related challenges.

## Data Availability

The datasets presented in this study can be found in online repositories. The names of the repository/repositories and accession number(s) can be found at: https://www.ncbi.nlm.nih.gov/genbank/, PRJNA1127609. The KBase narrative containing the genome assemblies and annotation tools run on the assemblies are also publicly available at narrative.kbase.us/narrative/135275.
