# Supplementary material for: A stable 15-member bacterial SynCom promotes Brachypodium growth under drought stress
Source: Front Microbiol. 2025 Aug 11;16:1649750. doi: 10.3389/fmicb.2025.1649750 (PMC12375656; doi:10.3389/fmicb.2025.1649750)
Supplement: Supplementary file 1 [file Data_Sheet_1.zip › Supplementary Figures.docx]

**Supplementary figures**

**
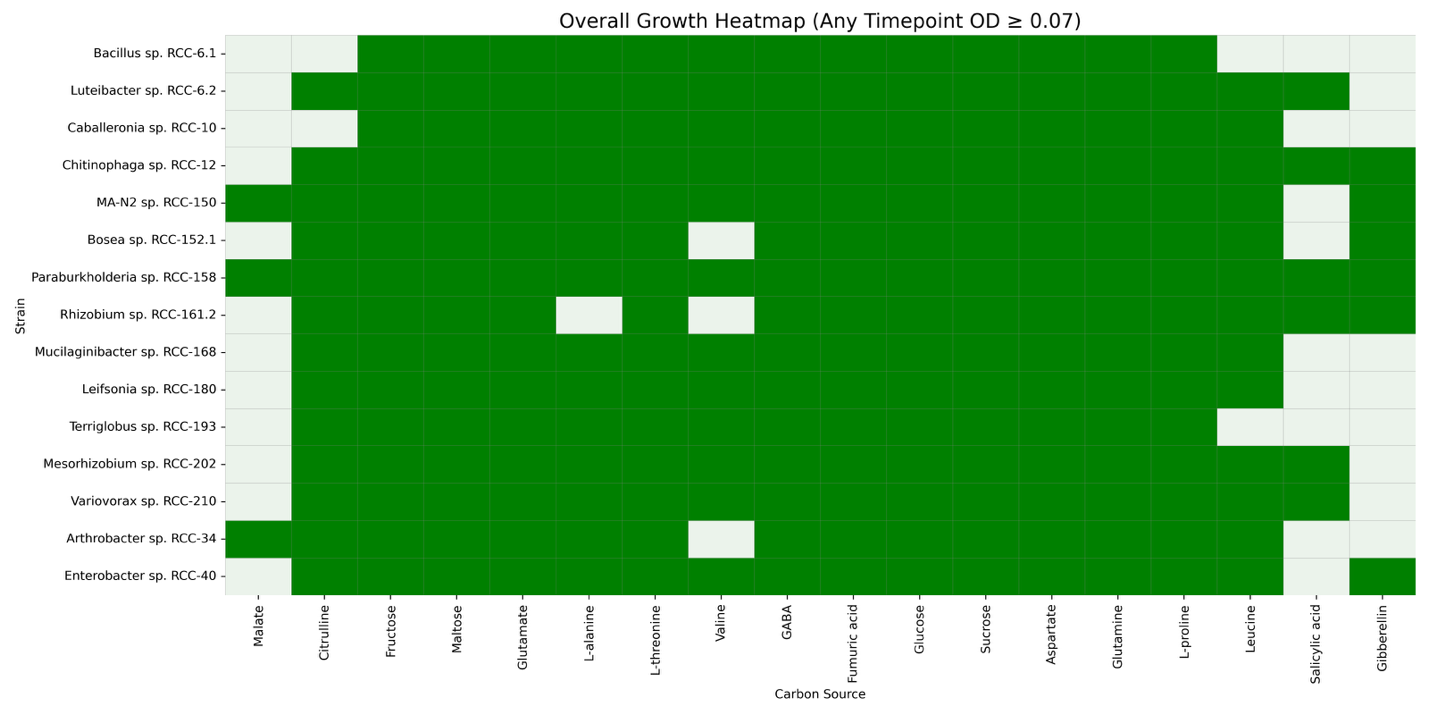
**

**Supplementary Figure S1:** Heatmap showing in vitro growth of the 15 SynCom isolates on 18 drought-enriched Brachypodium root exudate compounds. Each tile is shaded green if the strain achieved an OD600 increase ≥0.07 at any timepoint (24–168 h) on that carbon source, and light gray if it did not. Carbon sources (columns) include key osmoprotectants and TCA intermediates (malate, citrate, fumarate), amino acids (proline, leucine, valine, etc.), sugars (glucose, sucrose, fructose, maltose), and metabolites linked to drought stress (GABA, salicylic acid, gibberellin).

**
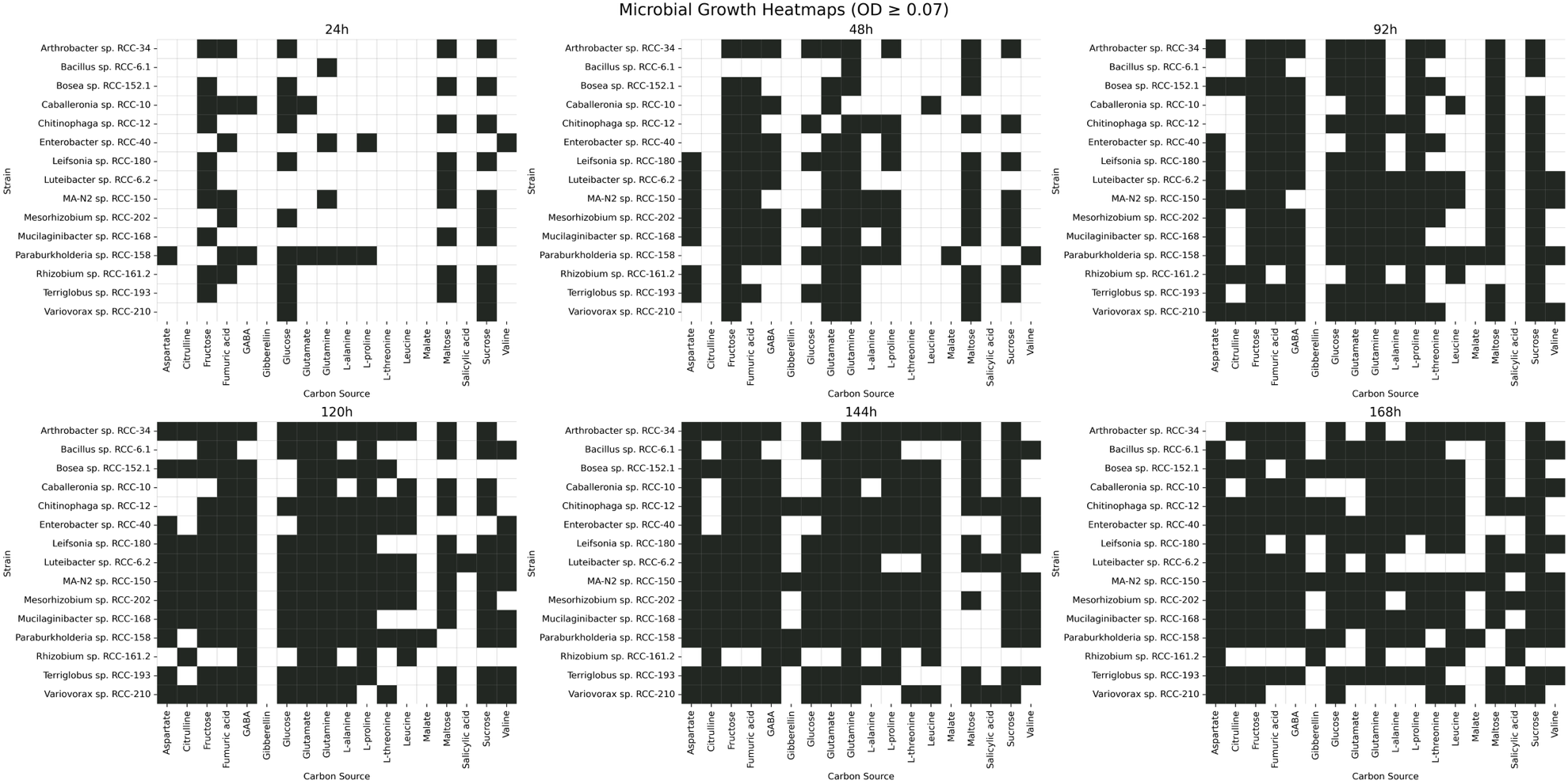
**

**Supplementary Figure S2:** Multi‐panel heatmaps of in vitro growth (OD600 ≥ 0.07) for the 15 SynCom isolates on 18 drought-enriched Brachypodium exudate compounds across six timepoints (24, 48, 92, 120, 144, and 168 h). Each panel shows strains (rows; ordered phylogenetically from Arthrobacter to Variovorax) versus carbon sources (columns; including TCA intermediates, amino acids, sugars, and stress-linked metabolites). Black tiles indicate a significant OD600 increase (≥0.07) at that timepoint on a given substrate, while white tiles denote no detectable growth.


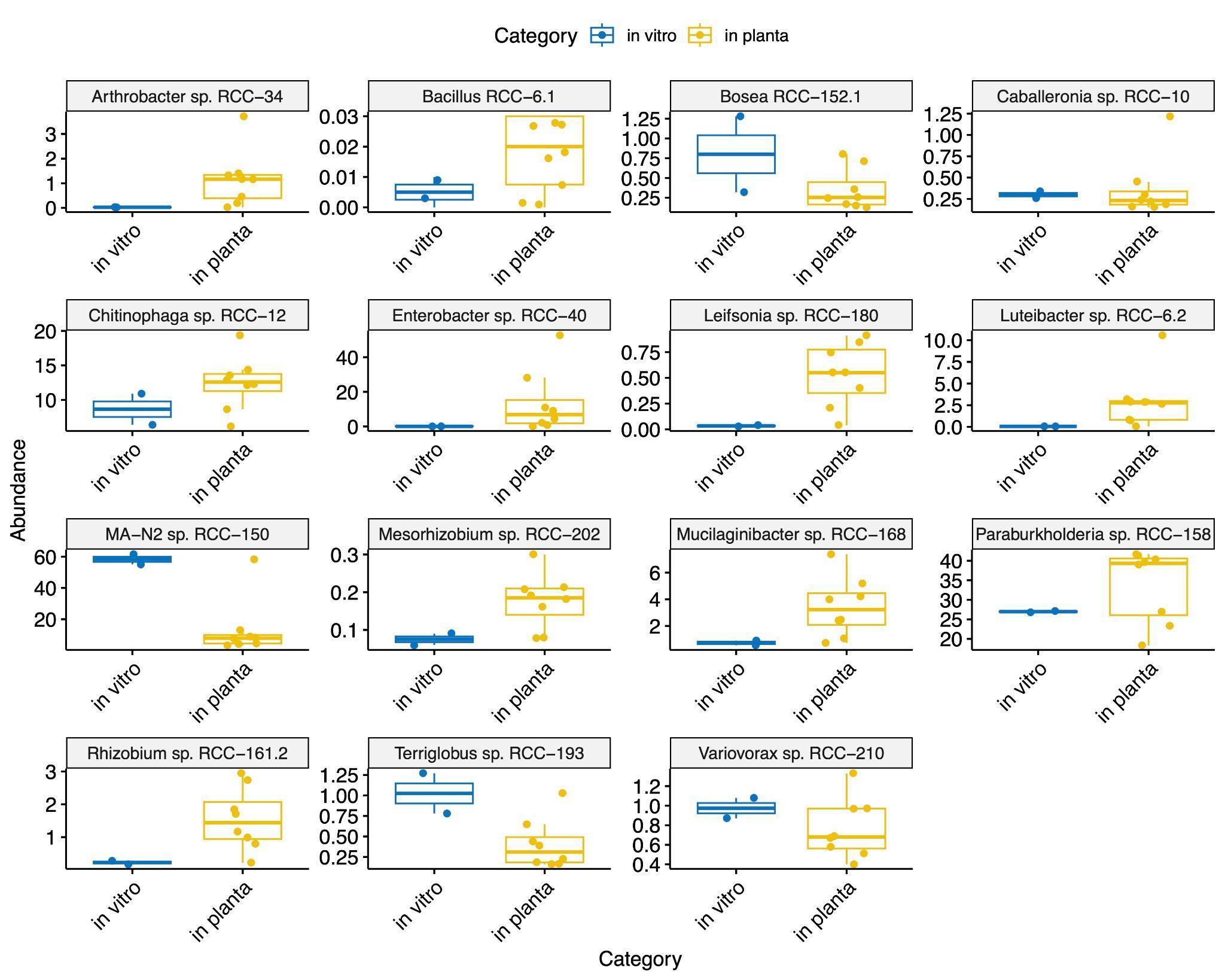


**Supplementary Figure S3:** Boxplot illustrating the relative abundance and distribution of SynCom members from both *in vitro* and *in planta* experiments, as determined by 16S rRNA gene analysis. This plot emphasizes the stability of SynCom member abundances, regardless of the presence of the rhizosphere. The x-axis represents the experimental treatment, with *in planta* in yellow and *in vitro* in blue, while the y-axis depicts the percentage of relative abundance at the end of the experiment. Each box corresponds to one of the 15 SynCom members, labeled at the top of the box and each dots represents samples.

Day 0: Seedlings transferred to the pots


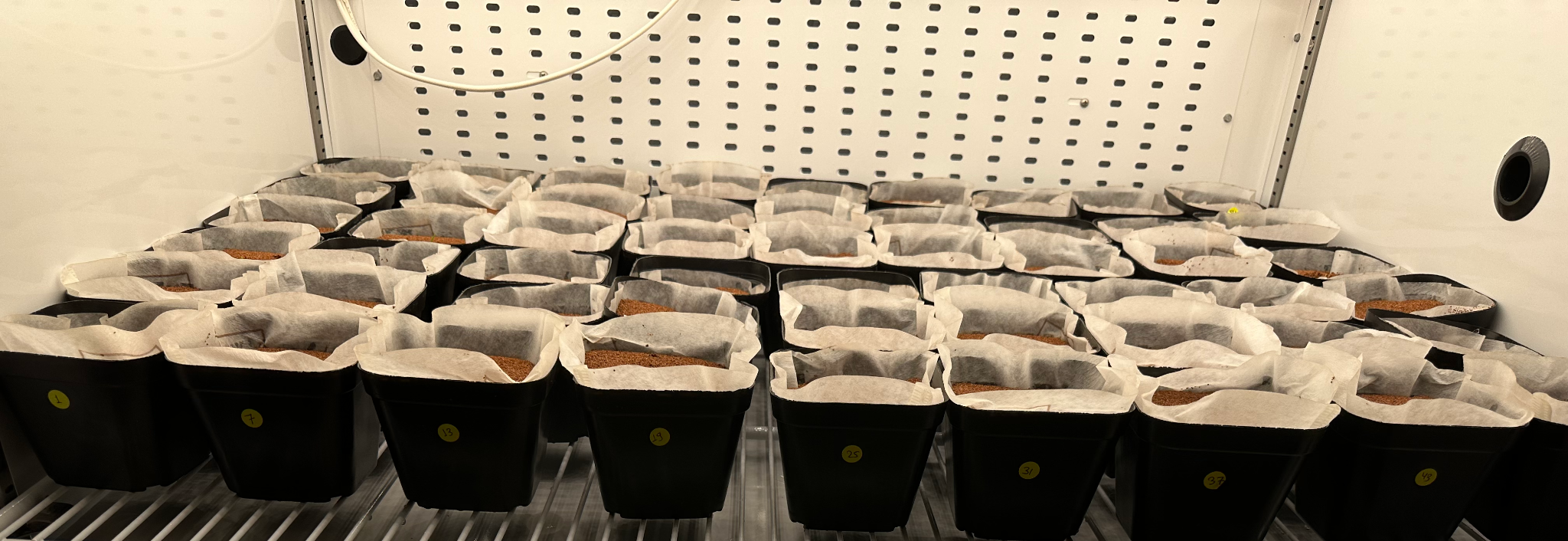


Day7: Seedlings grown in the pot for a week


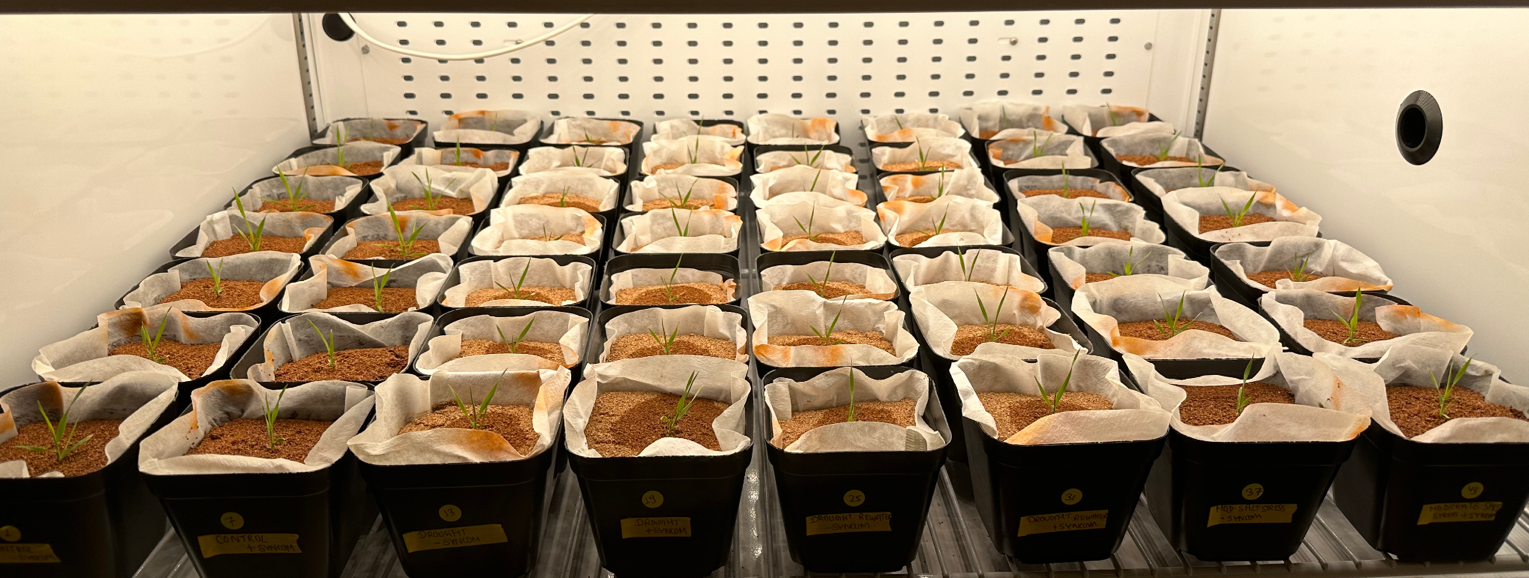

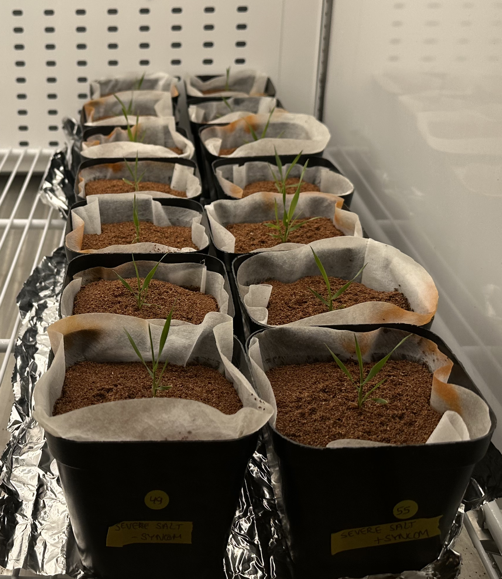


Day 21: Seedlings grown in the pot for 3 weeks before sampling


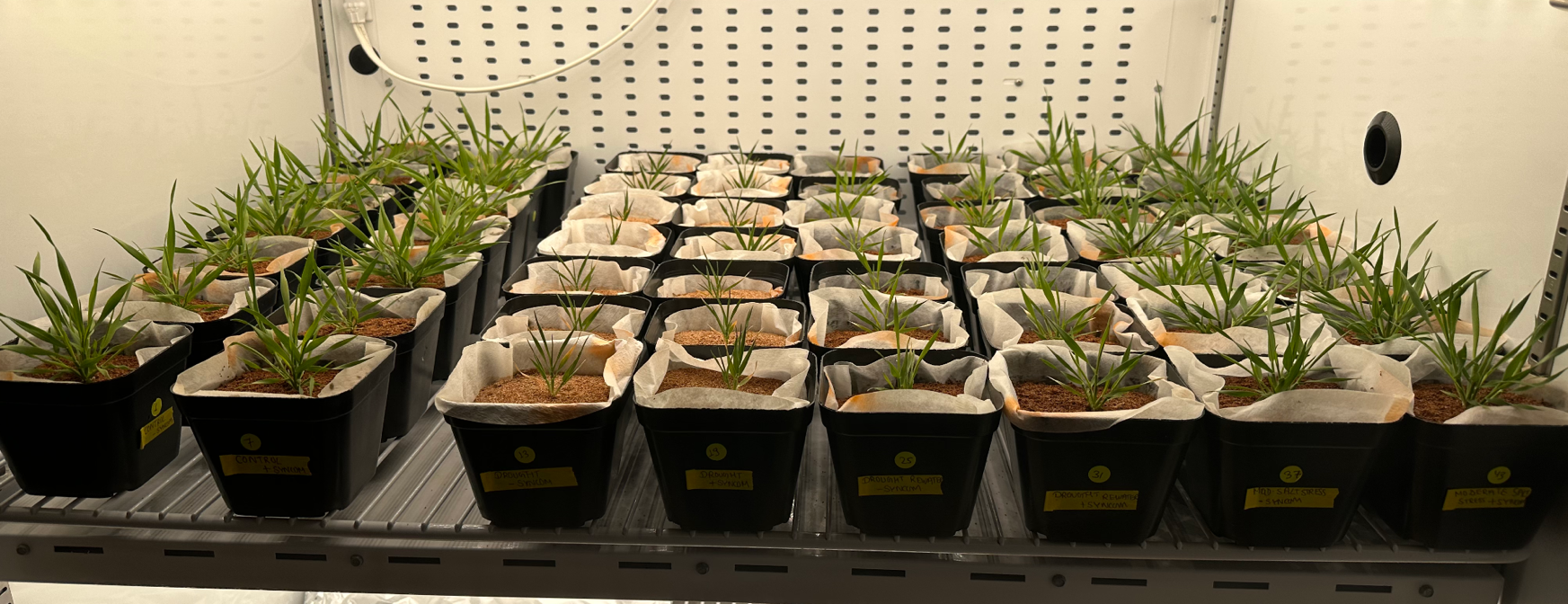

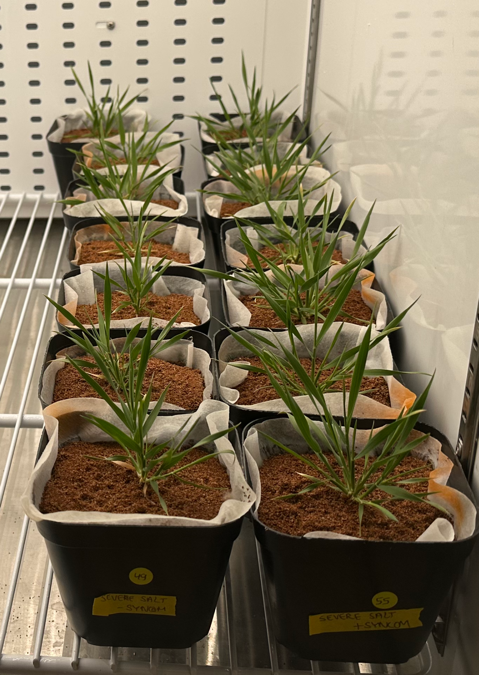


**Supplementary Figure S4:** Photographs of the pots used in the plant experiment, taken on Day 0, Day 7, and Day 21 (sampling day). Each column of 7 pots (replicates) represents a different treatment. From left to right, the treatments are as follows: control (No SynCom), control (With SynCom), drought (No SynCom), drought (With SynCom), rewatered drought (No SynCom), rewatered drought (With SynCom), low salt stress (With SynCom), low salt stress (No SynCom), salt stress (With SynCom), and salt stress (No SynCom). Please note that the low salt stress results are not included in this manuscript.

**Supplementary Figure S5**: Functional redundancy of SynCom isolates calculated using available PGP traits annotated from their genomes. The x-axis represents the inoculum and different *in planta* conditions such as control and stress conditions including drought, rewatered drought, and salt. The y-axis represents the functional redundancy (FR) values estimated using the R package “SYNCSA”. The asterisk indicates significantly different functional redundancy compared to the control.


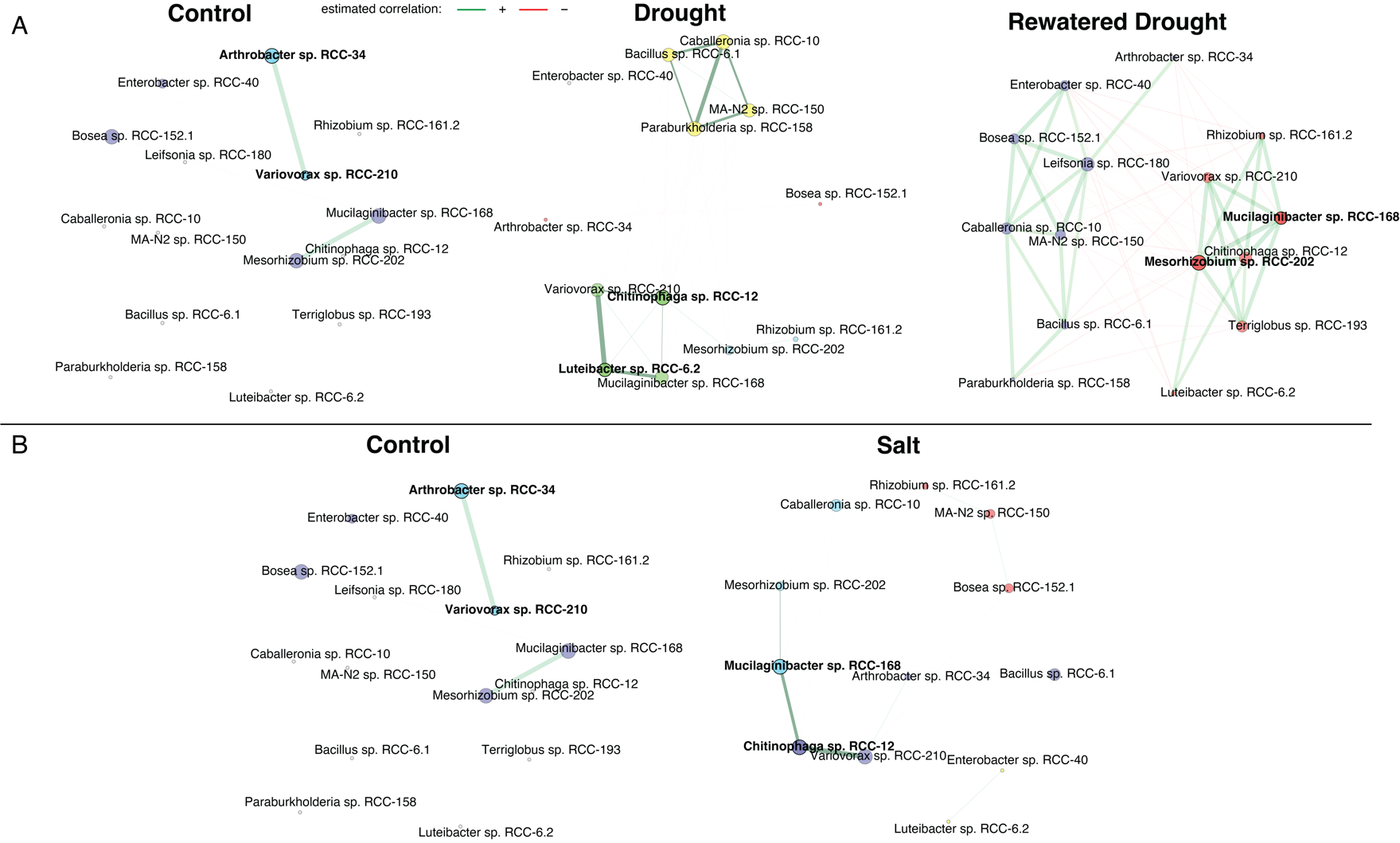


**Supplementary Figure S6:** Comparison of bacterial association networks between rhizosphere samples under different experimental conditions (A) control versus drought, and (B) control versus salt. R package “NetCoMi” was used to construct and visualize the network. Networks were generated using the fast greedy clustering algorithm with a Pearson correlation coefficient threshold of ± 0.3, and a t-test (< 0.05) for sparse matrix generation. Eigenvector centrality (>0.90) is used for defining hubs and scaling node sizes. Node colors represent clusters and clusters have the same color in both networks if they share at least two taxa. Green edges correspond to positive estimated associations and red edges to negative ones. Hubs are highlighted with bold boundaries and corresponding isolate names.

Set: “Control”


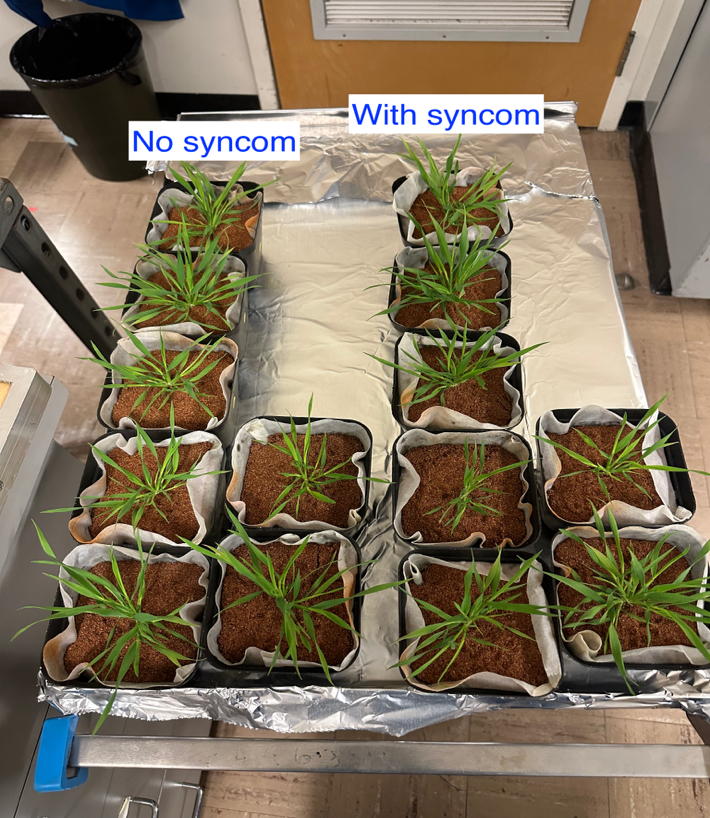

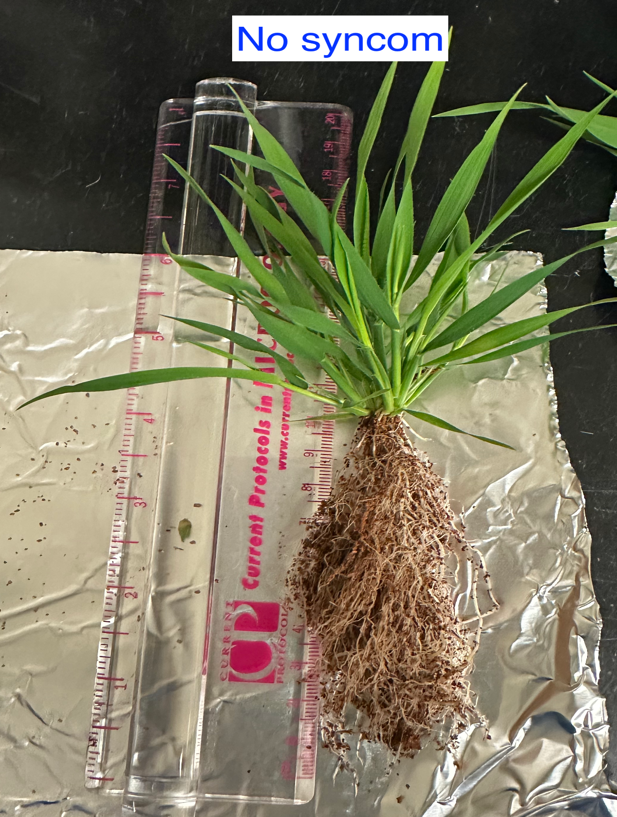

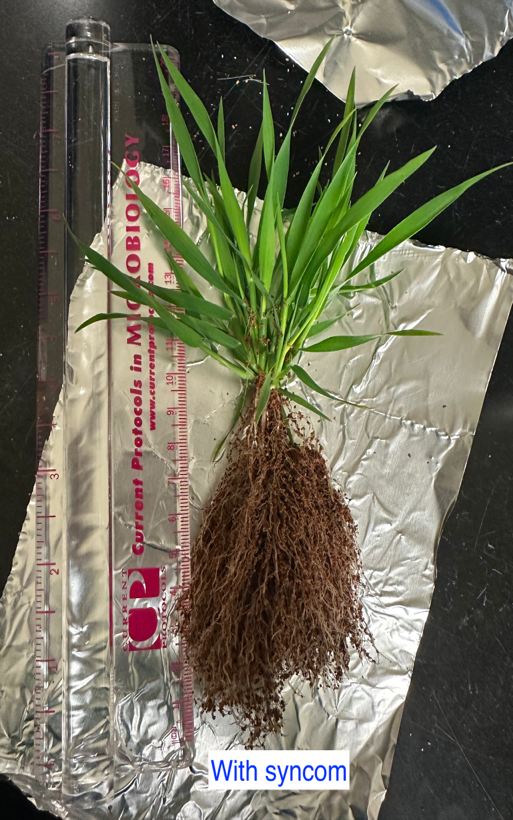


Set: “Drought”


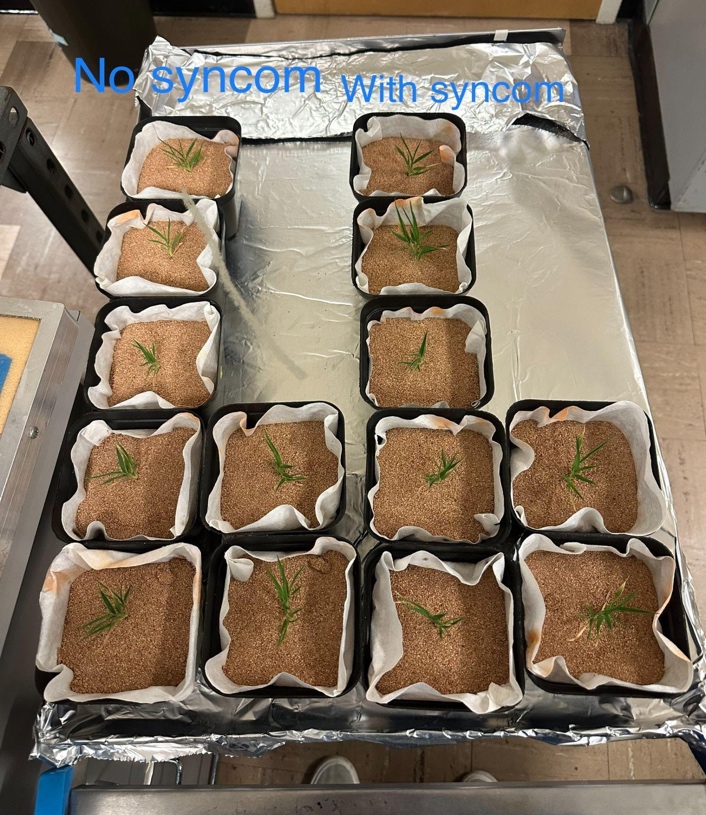

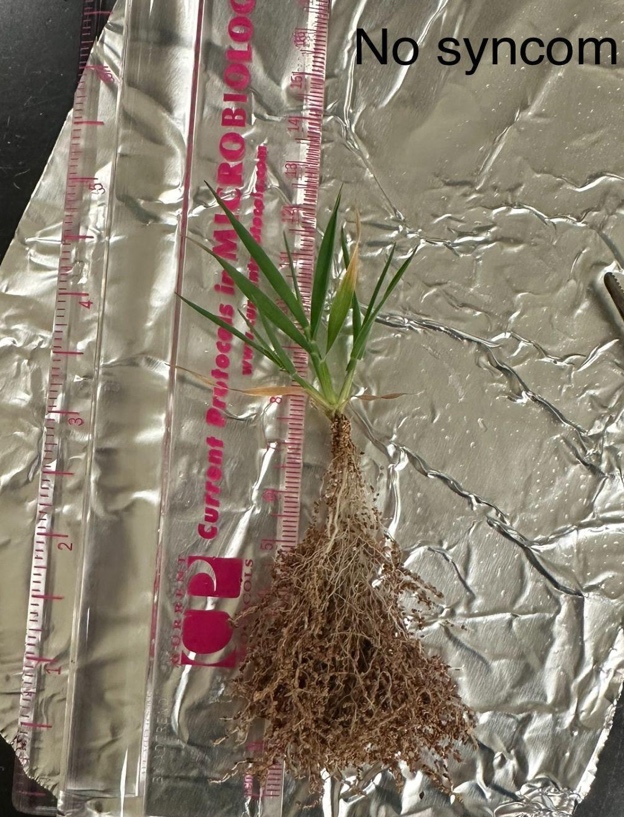

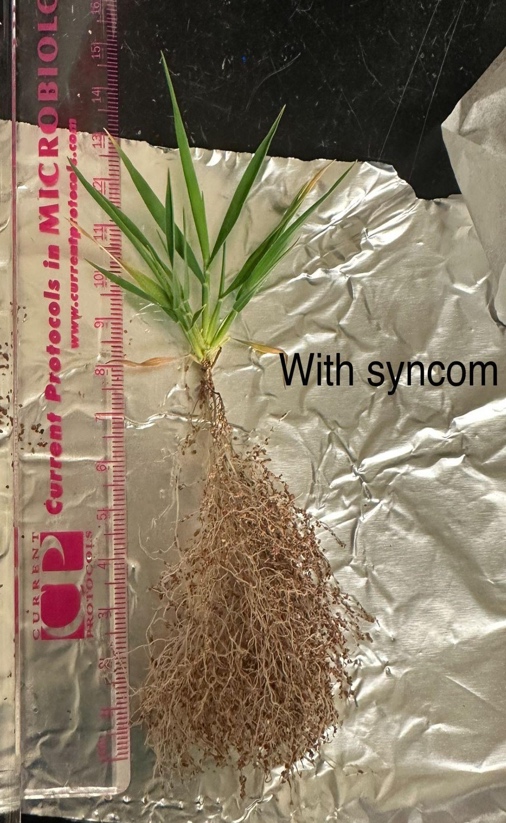


Set: “Rewatered”


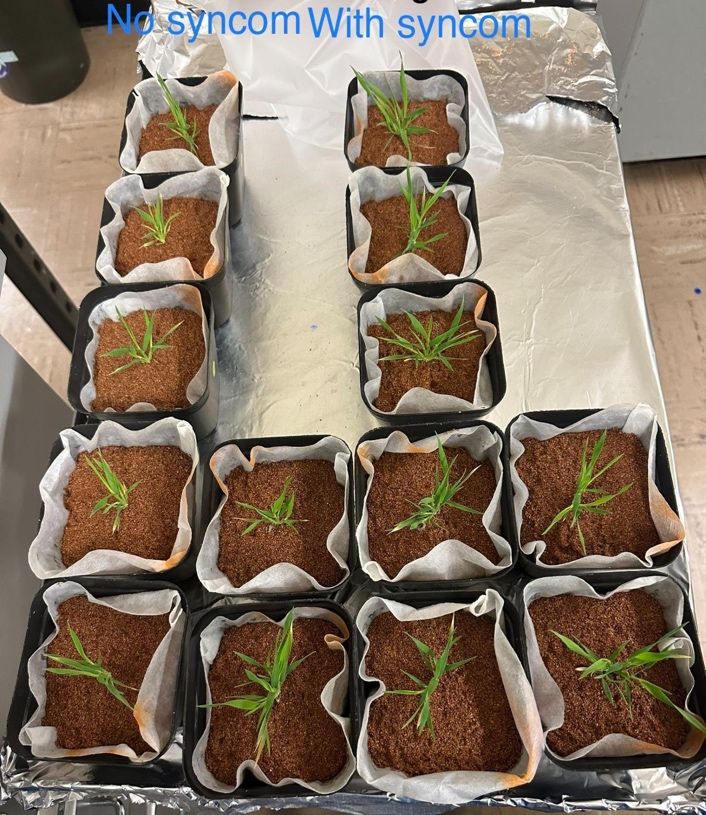

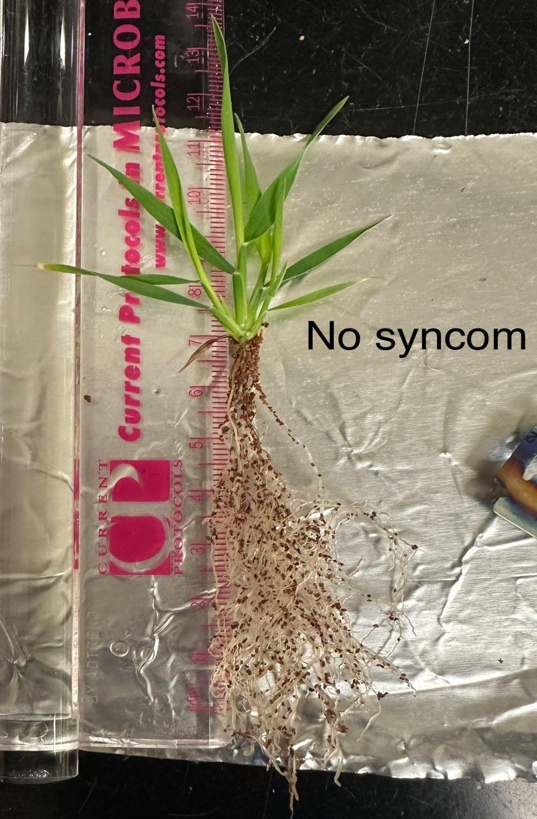

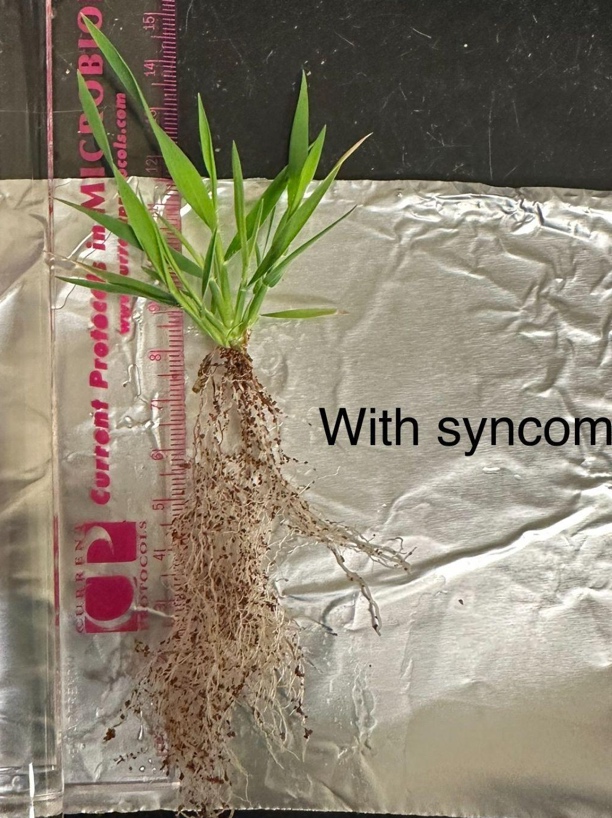


**Supplementary Figure S7:** Plant performance images for "Control" and "Drought" plants. The first image in

each set shows all seven replicates of plants for both the "with SynCom" and "without SynCom" groups. The

two images on the right provide a comparison of these groups for a randomly selected plant from each category.

RCC-6.1 RCC-6.2


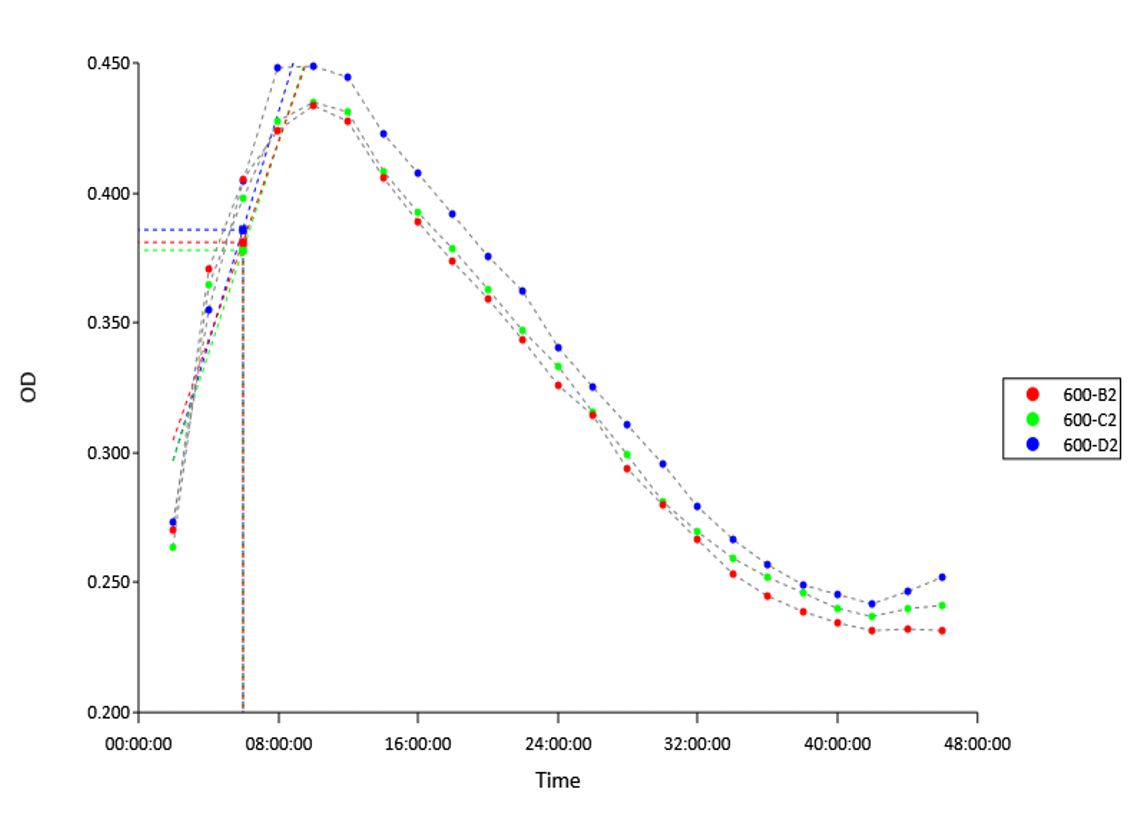

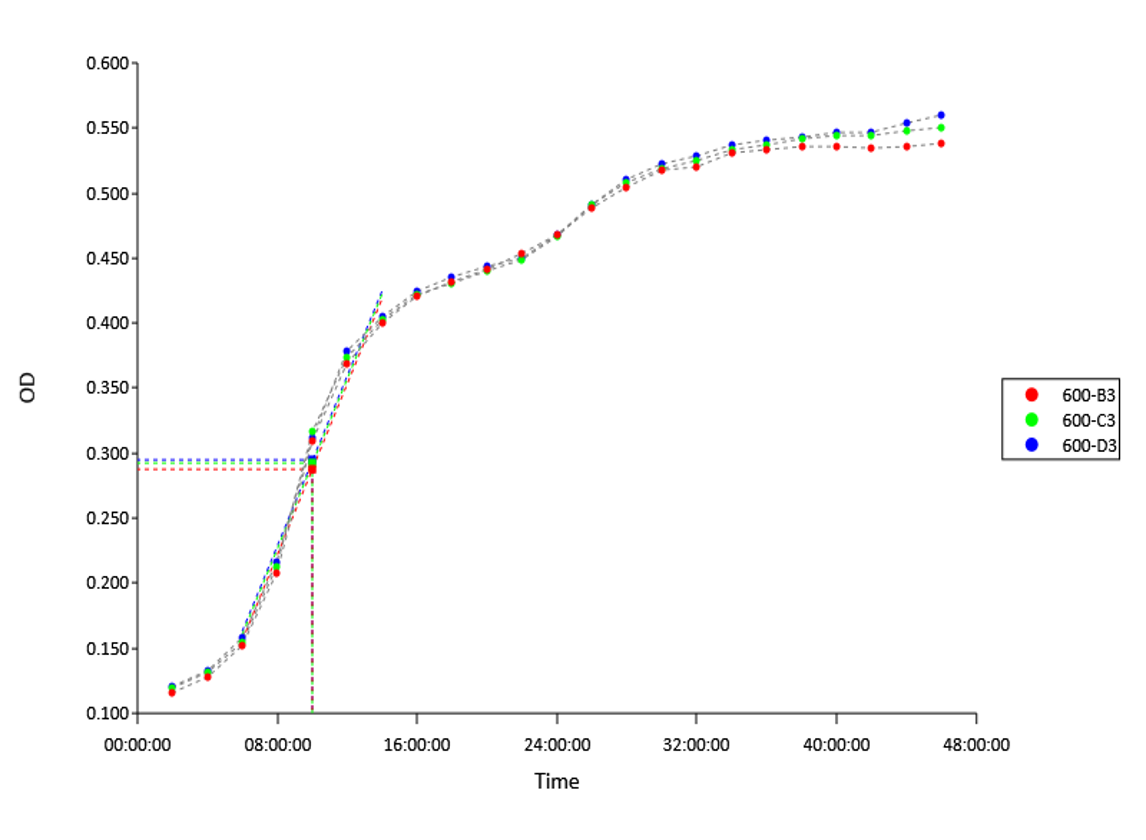


RCC-10 RCC-12


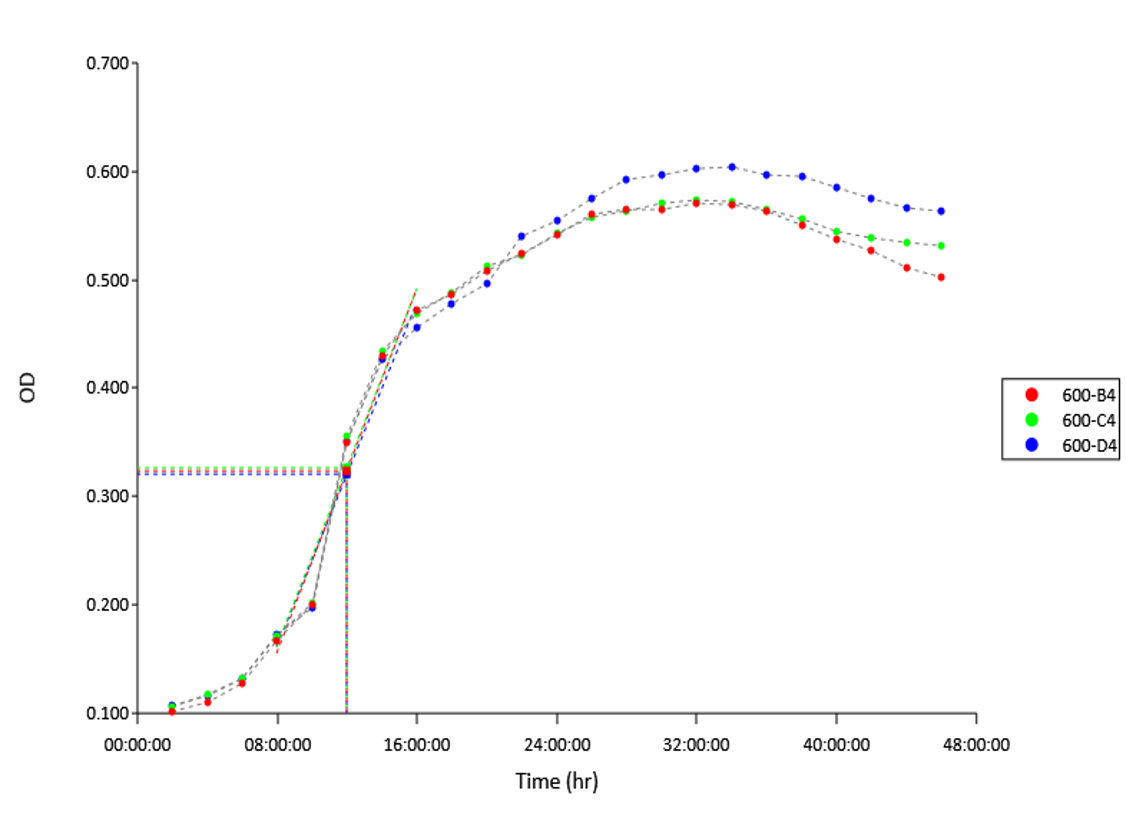

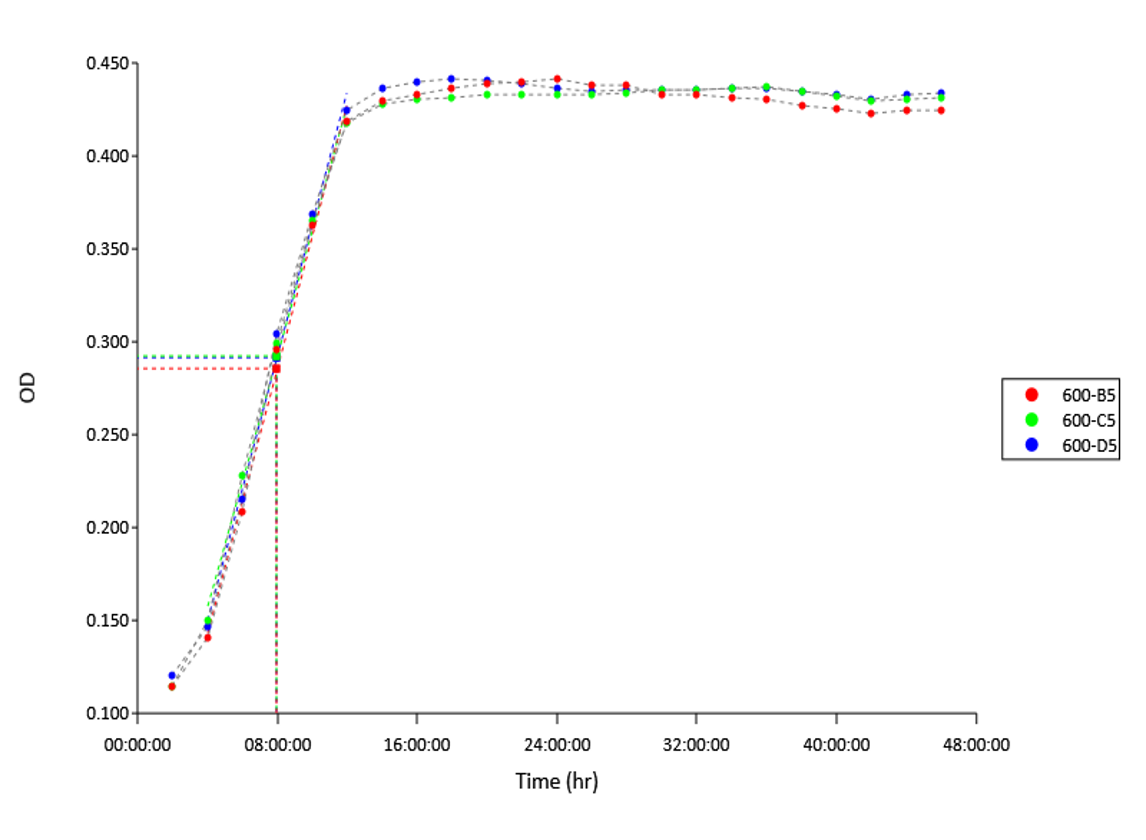


RCC-34 RCC-40


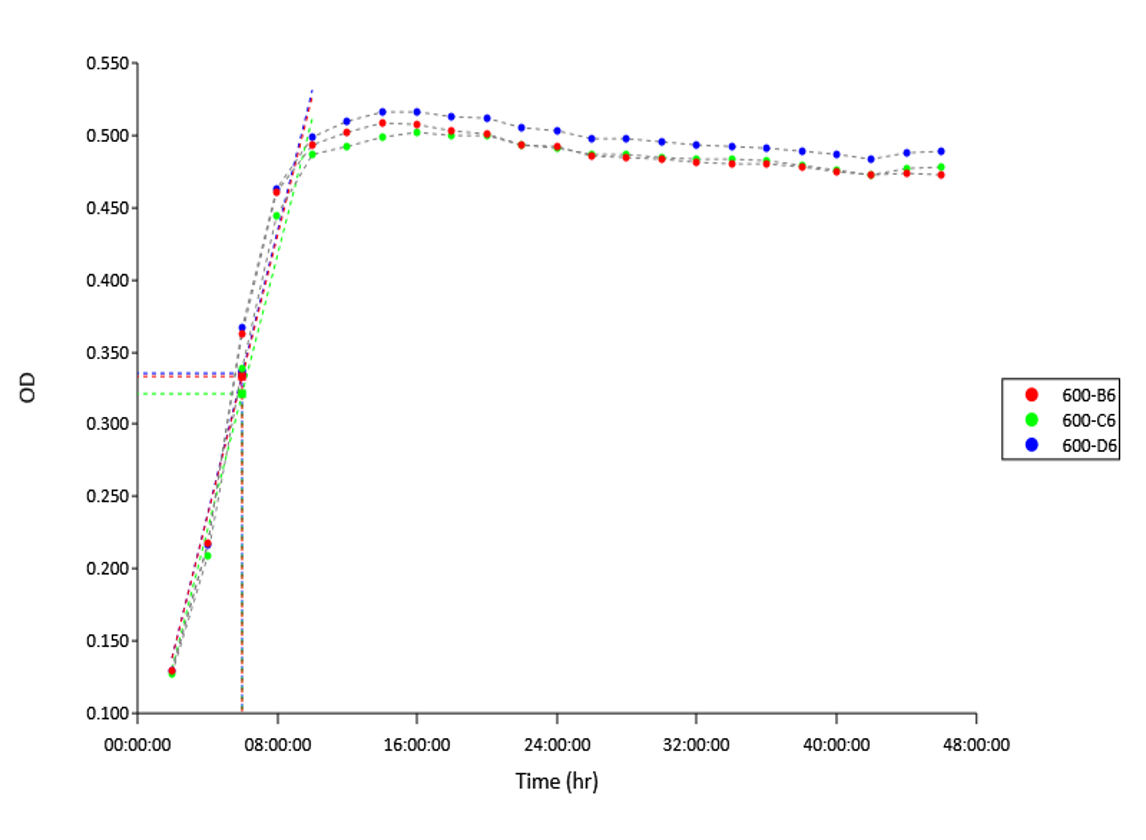

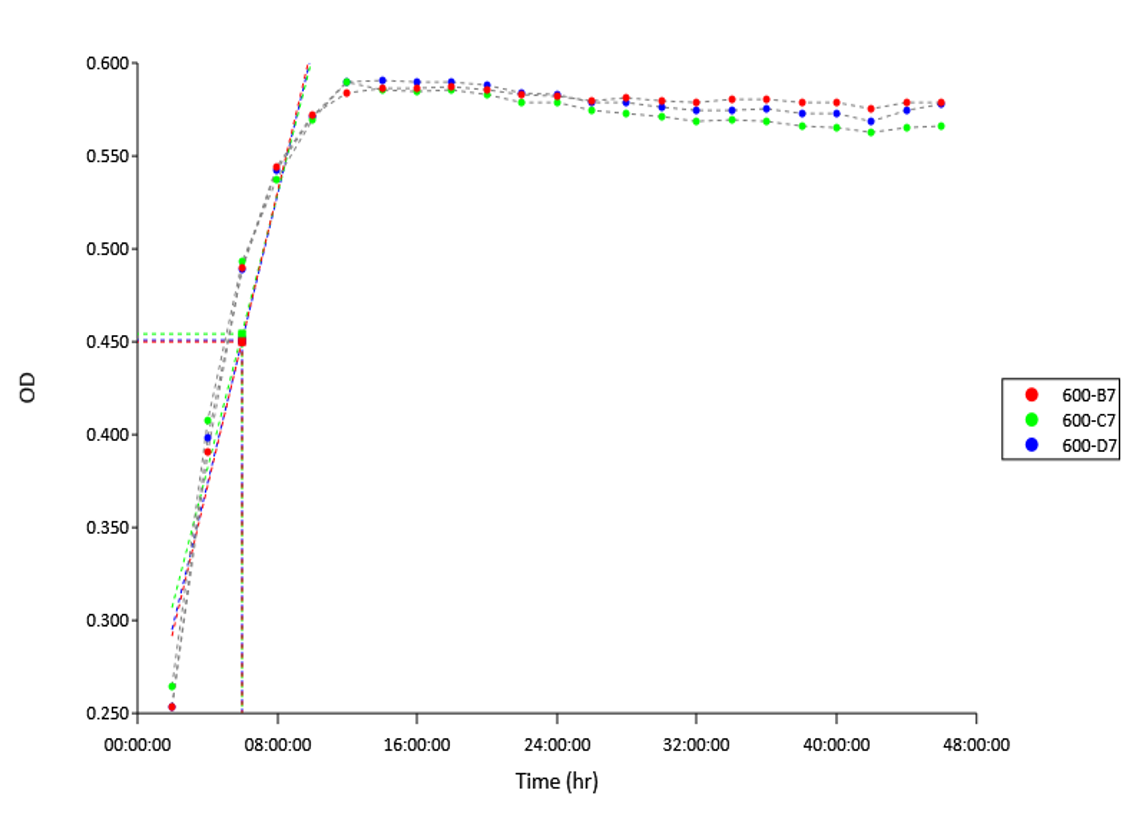


RCC-150 RCC-152.1


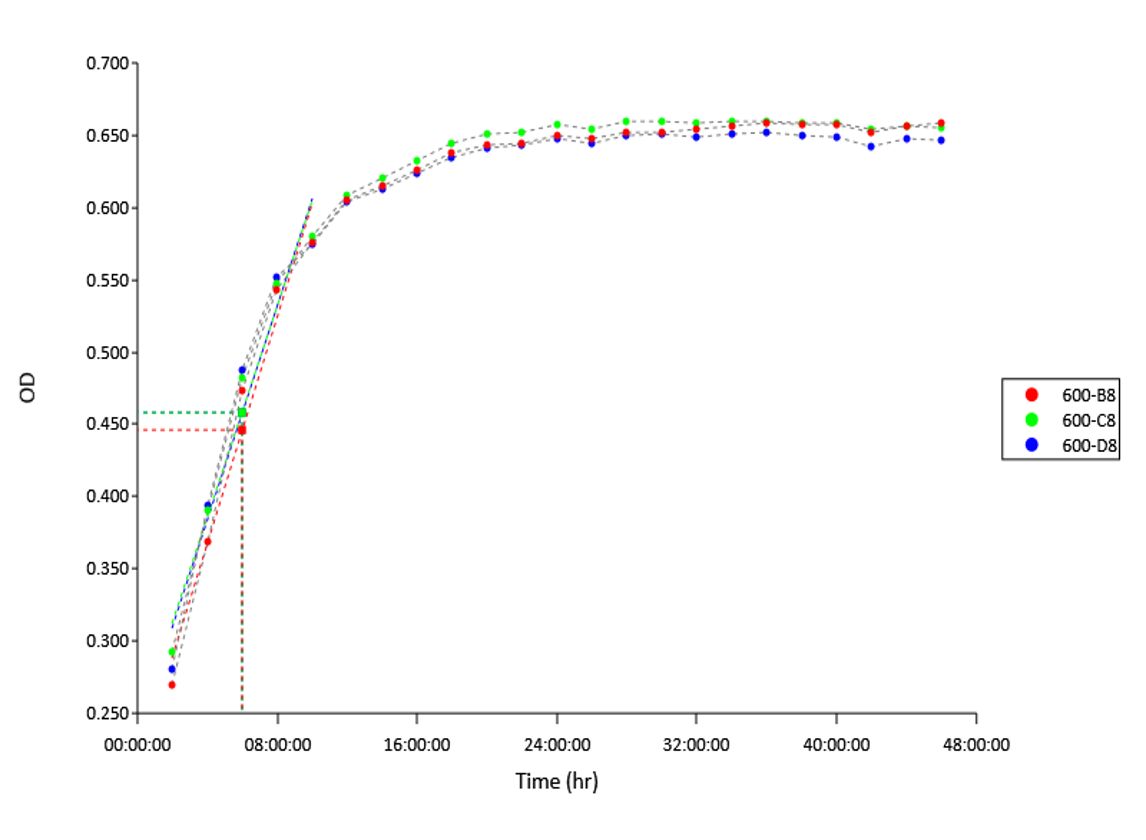

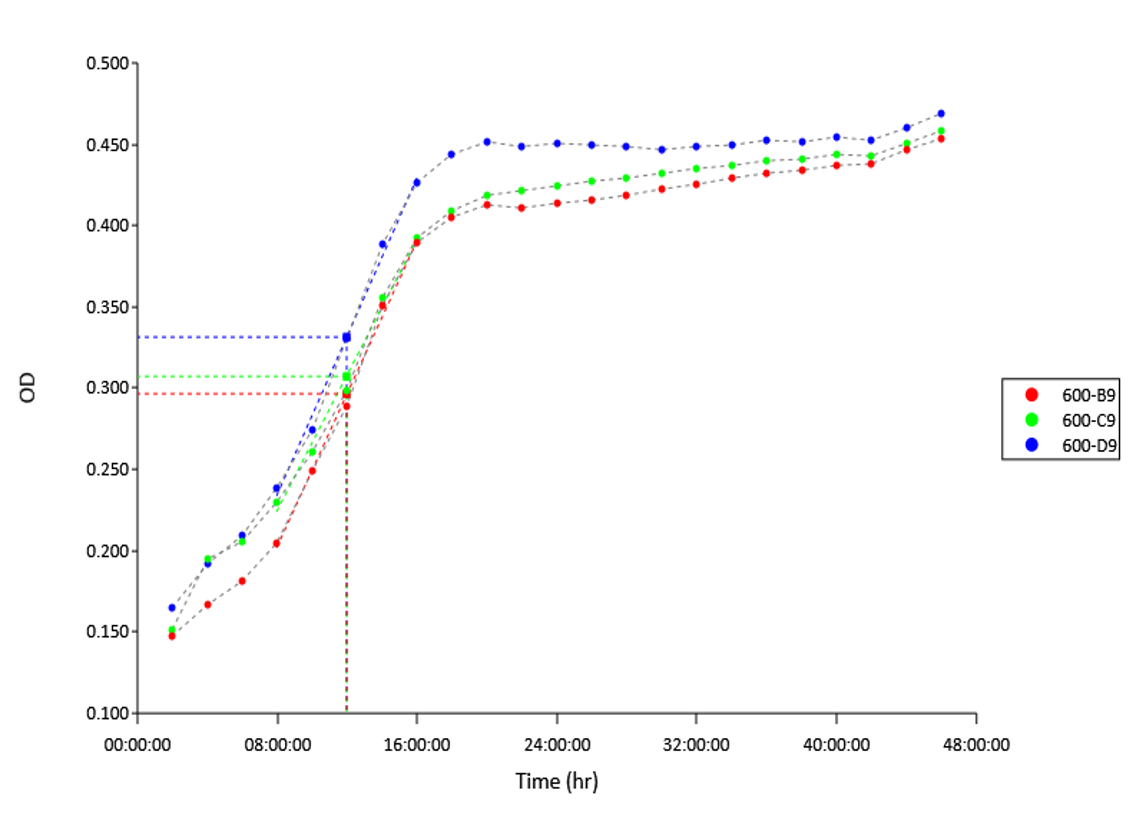


RCC-158 RCC-161.2


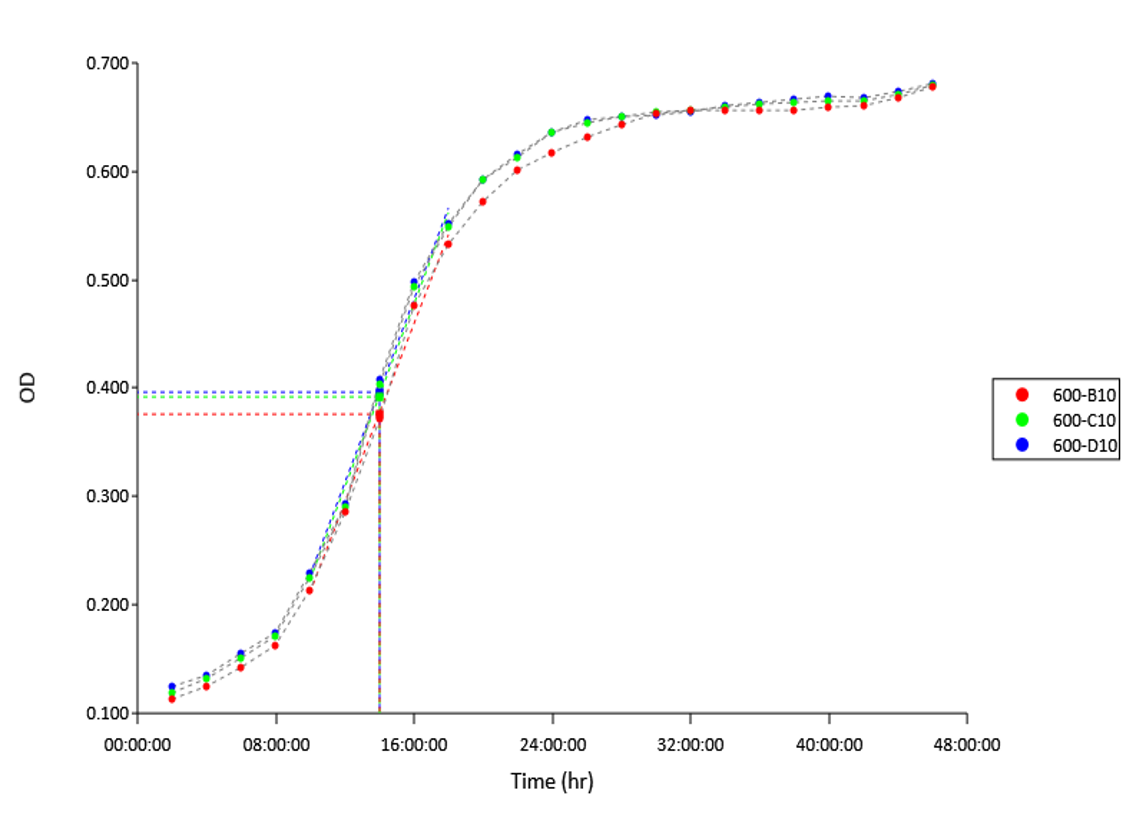

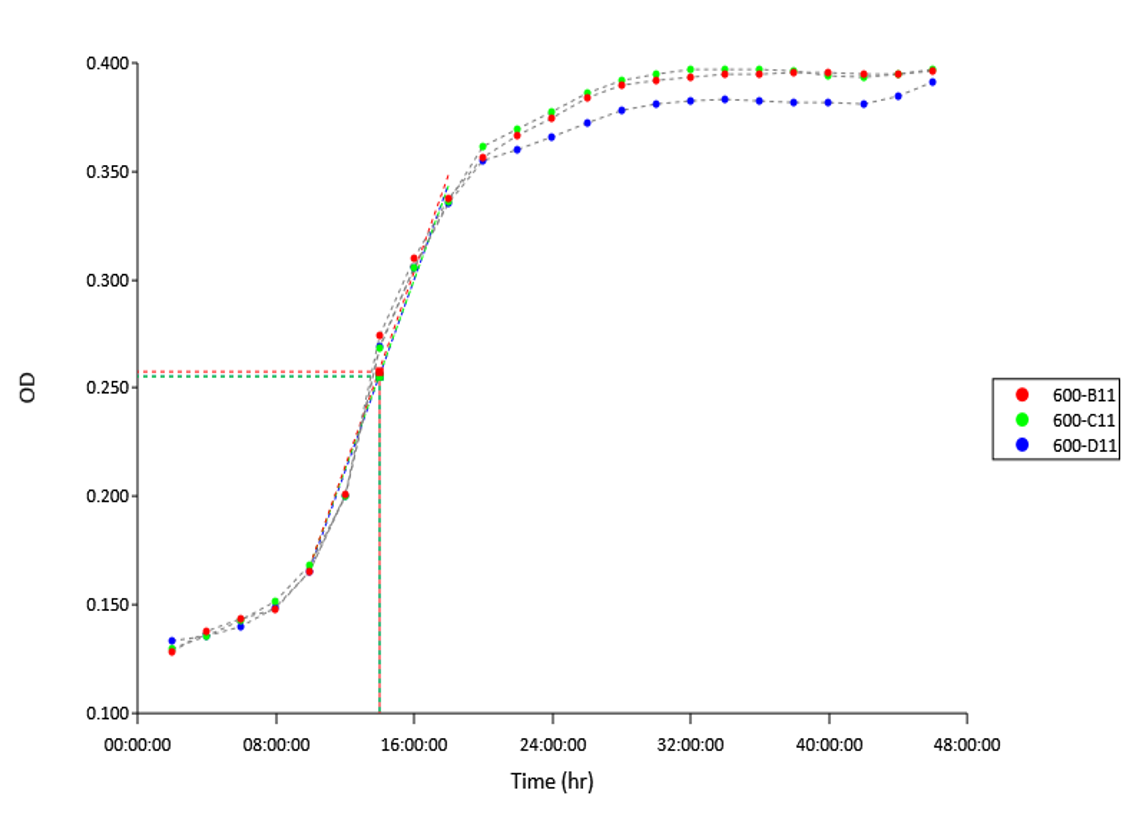


RCC-202 RCC-210


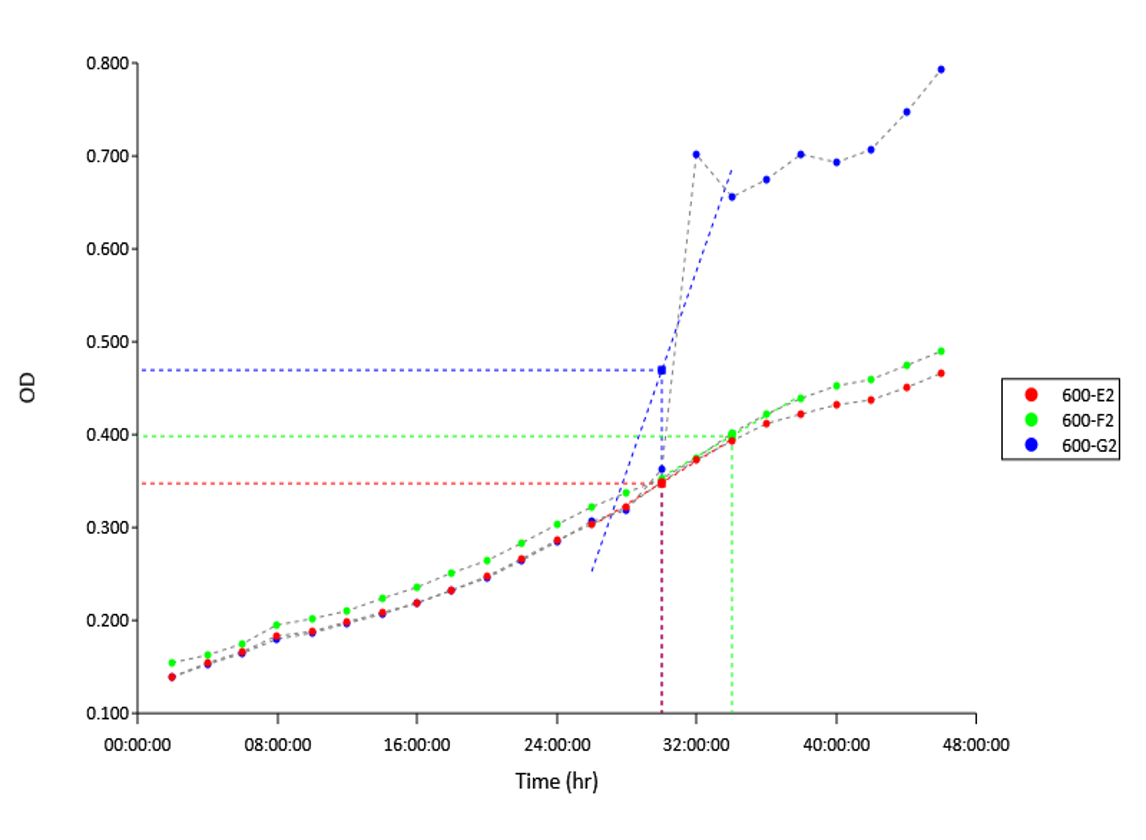

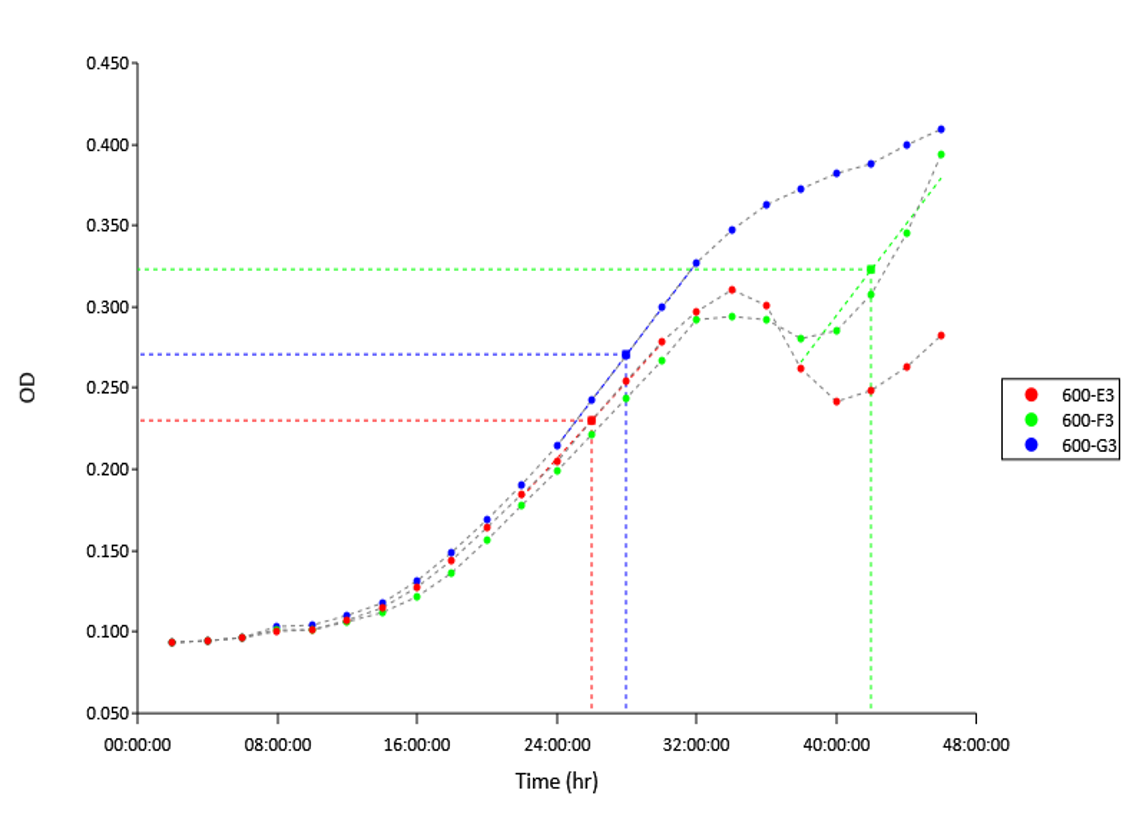


RCC-193 RCC-180


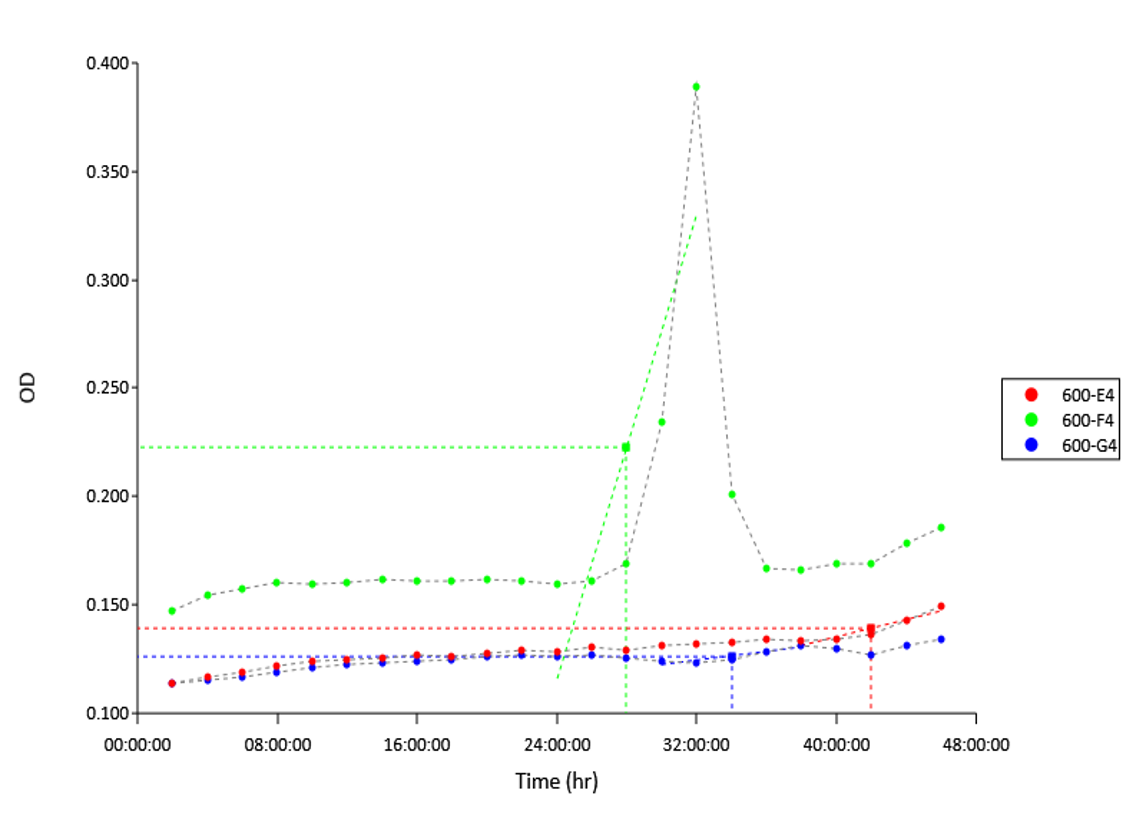

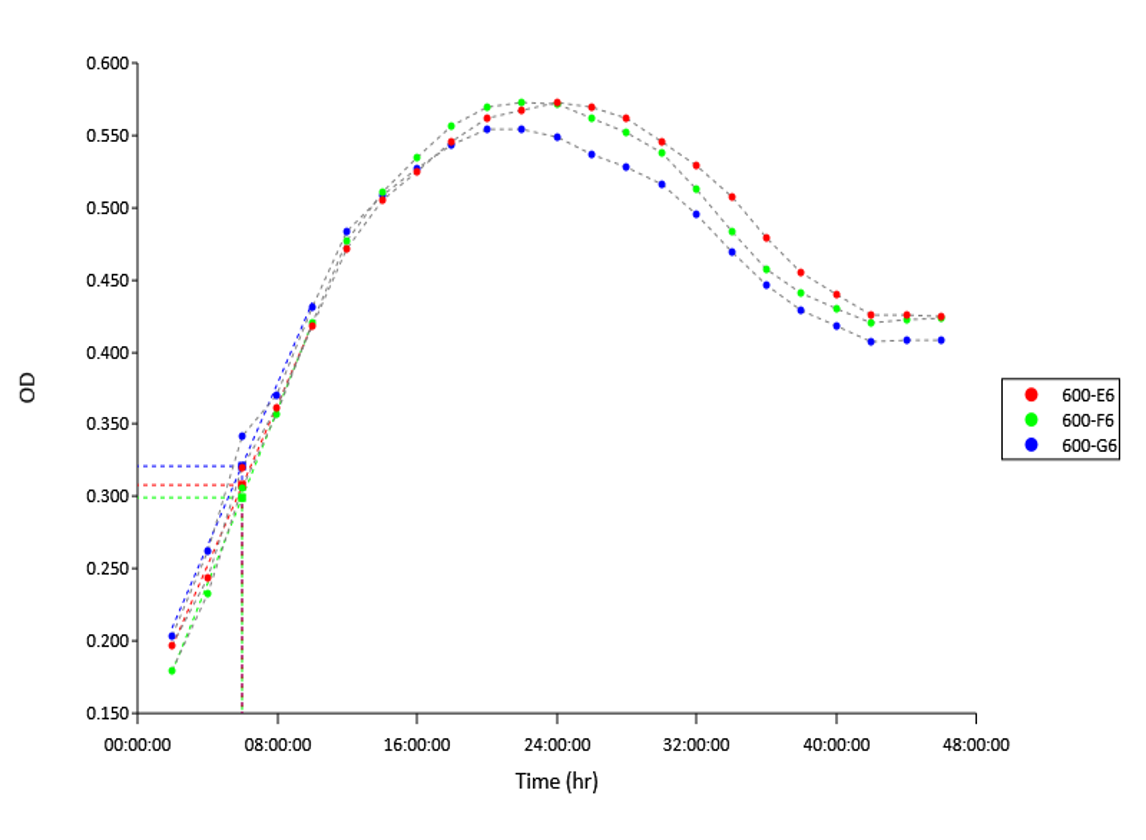


RCC-168


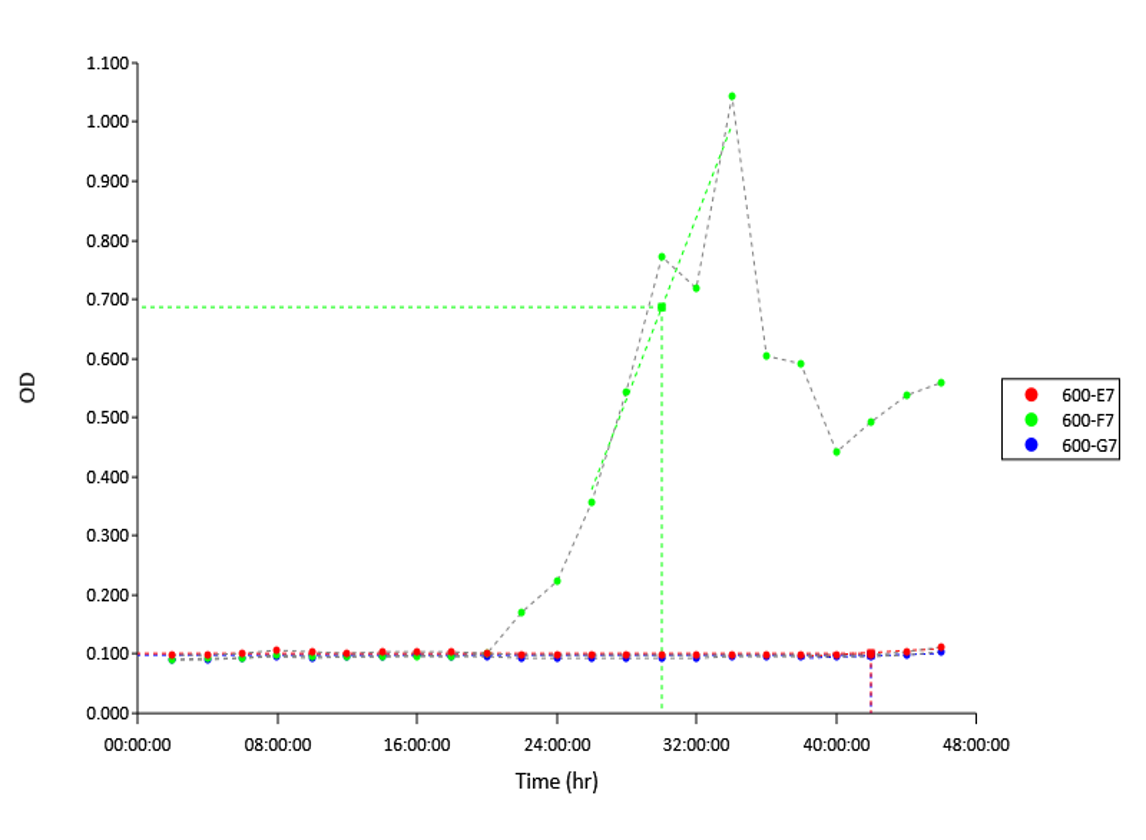


**Supplementary Figure S8:** Growth curve for the 15 Syncom isolates (triplicates for each isolates) when grown in R2A media.
